# Mechanisms upholding the persistence of stigma across 100 years of historical text

**DOI:** 10.1038/s41598-024-61044-z

**Published:** 2024-05-14

**Authors:** Tessa E. S. Charlesworth, Mark L. Hatzenbuehler

**Affiliations:** 1 Kellogg School of Management, Northwestern University, 2211 Campus Dr, Evanston, IL 60208 USA; 2 Department of Psychology, Harvard University, 33 Kirkland St, Cambridge, MA 02138 USA

**Keywords:** Stigma, Stereotypes, Historical change, Text analysis, Word embeddings, Human behaviour, Health policy

## Abstract

Today, many social groups face negative stereotypes. Is such negativity a stable feature of society and, if so, what mechanisms maintain stability both within and across group targets? Answering these theoretically and practically important questions requires data on dozens of group stereotypes examined simultaneously over historical and societal scales, which is only possible through recent advances in Natural Language Processing. Across two studies, we use word embeddings from millions of English-language books over 100 years (1900–2000) and extract stereotypes for 58 stigmatized groups. Study 1 examines aggregate, societal-level trends in stereotype negativity by averaging across these groups. Results reveal striking persistence in aggregate negativity (no meaningful slope), suggesting that society maintains a stable level of negative stereotypes. Study 2 introduces and tests a new framework identifying potential mechanisms upholding stereotype negativity over time. We find evidence of two key sources of this aggregate persistence: within-group “reproducibility” (e.g., stereotype negativity can be maintained by using different traits with the same underlying meaning) and across-group “replacement” (e.g., negativity from one group is transferred to other related groups). These findings provide novel historical evidence of mechanisms upholding stigmatization in society and raise new questions regarding the possibility of future stigma change.

## Introduction

Stigma—defined as the co-occurrence of labeling, stereotyping, separation, status loss, and discrimination in a context in which unequal power is exercised^1^—is a central topic throughout social science^2–6^. Robust evidence indicates that stigmatization affects the distribution of life outcomes (e.g., health, employment, educational attainment) for a diverse range of statuses, such as race, ethnicity, sexuality, disability, religion, immigration, and mental (e.g., schizophrenia) and physical (e.g., HIV) health conditions^7^. Both the number of targeted groups and the adverse consequences of stigmatization have motivated extensive efforts to understand whether, when, and how stigma might be reduced^8^.

Social science theories suggest that such efforts to reduce stigma might face serious challenges because stigmatization serves multiple evolutionary, psychological, and social functions—including to avoid perceived pathogens^9^, to justify and maintain the status quo^10^, and to exploit and dominate others for political and economic gains^11^. These theories raise the provocative hypothesis that societies maintain a relatively stable level of stigmatization, in which the aggregate level of stigma across many groups remains flat over time. By aggregate, we mean the average level of stereotype negativity in society, observed by averaging trends across a diverse sample of stigmatized groups. Moreover, if aggregate persistence is observed, it raises new questions regarding the mechanisms underlying such stability. The current project provides the first historical test of both questions. Specifically, we ask: Is stigmatization—as revealed through aggregating trends in collective stereotypes towards 58 diverse groups—a stable feature of society? And if so, what mechanisms maintain stability of negativity both within and across groups?

Existing research on questions of stigma stability or change, as well as the underlying mechanisms upholding such patterns, typically relies on archival data capturing components of stigma (e.g., stereotypes) that scientists happened to collect in past surveys and experiments^12^. While such approaches have provided important insights, they nevertheless remain limited to understanding stigma change as it unfolds: (a) towards one group target (or, in rare cases, a small subset of groups) studied in isolation; (b) over a short timescale, typically days or weeks; and (c) at the individual level of analysis (e.g., respondents measured pre/post interventions).

Yet to address whether and how stigma is stable on aggregate in society, we require a very different approach. First, because our question is about the aggregate level of stigma, it can only be examined by averaging the trends in stereotypes across *dozens* of stigmatized groups that represent a wide sample space of group targets. Additionally, as we elaborate below, a wide sample space of group targets is also critical for testing whether and how stereotype negativity may transfer or generalize between groups over time to uphold aggregate negativity across groups. Second, by definition, examining persistence requires a long-term, historical dataset that studies these diverse groups simultaneously over a sufficiently long period (ideally, multiple decades) to allow for the detection of any reasonable change, should it exist. And finally, our question about stigma at a societal level necessitates methods that explicitly seek to examine collective, shared representations rather than a single individuals’ endorsed attitudes.

Drawing on recent advances in Natural Language Processing (NLP)^13^, the current work created a new historical dataset that meets these requirements to address multi-group, long-term, societal-level change in stigma. Specifically, as elaborated in *Methods* below, we use word embeddings trained on Google Books from 1900 to 2000^14^ (and replicate all analyses in the Corpus of Historical American English). Word embeddings utilize word co-occurrences to quantitatively represent word meaning as vectors. The result is that words that tend to co-occur in similar contexts (e.g., “woman” co-occurs with “home” or “kind” more than “office” or “assertive”) will have vectors that are closer together in space. Thus, relationships between vectors can be used to identify stereotypical associations (e.g., between “woman” and “home”)^15,16^. Already, researchers have demonstrated the validity of studying stereotype change in word embeddings, showing that the embeddings capture known changes in gender stereotypes alongside the Women’s movement^17,18^ as well as shifts in Asian stereotypes following immigration waves^18^. Most recently, these approaches have also been used to provide insights into *which* groups may change over time in historical text. Specifically, comparing stereotypes towards 36 stigmatized groups (and 36 non-stigmatized contrast groups), sociodemographic identities (i.e., demographic characteristics that are imbued with strong social significance such as race, religion, or ethnicity) were found to change more over 115 years than body-related identities (i.e., identities that are visible and physical such as body weight or disability)^19^.

Still, none of these projects have used a multi-group perspective focused on negatively stigmatized groups to test *aggregate* stereotypes at a societal level. Nor has any project yet considered the mechanisms upholding aggregate negativity both within groups (i.e., towards the same target) as well as across groups (i.e., towards multiple targets simultaneously). The novel contributions of the current manuscript are thus to: (1) examine patterns in aggregate negative stereotypes in society (Study 1); and (2) introduce and test a new theoretical framework that organizes a set of mechanisms underlying these trends (Study 2).

## Study 1: examining aggregate negative stereotypes across 100 years of text

Study 1 examines whether the aggregate negativity in stereotypes of stigmatized groups has either generally decreased, increased, or remained stable in English-language book text from 1900 to 2000. Past work would suggest that any of these three patterns are empirically and theoretically possible. First, a *decrease* in negativity might be expected given results from repeated cross-sectional surveys of explicit and implicit attitudes showing slow but steady drops in negative representations for some target groups between the early 2000s–2020^20–22^. Alternatively, *increasing* negativity might be expected, based on findings that as the number and visibility of several stigmatized groups increased over the past century, so too has the perceived threats of those groups, perhaps prompting negative backlash^23^. Indeed, recent history has seen rising hate crimes and legislation targeting stigmatized groups^24,25^. Finally, negativity may have remained *persistent* throughout the past 100 years. As reviewed above, some social science theories^9–11,26^ posit that stigmatization serves multiple evolutionary, psychological, and social functions. Thus, societies may maintain a relatively stable level of stigmatization because it allows individuals and groups to attain relevant goals.

## Results

For each of the 58 groups (represented by group label lists from historical thesauruses; Appendix), we ranked cosine similarities between the group and a list of 414 traits available across the 100 years (from a larger list of ~ 600 traits^27^). We then identified the top ten traits associated with each of the 58 groups in each decade. From these top-associated traits we also extract our primary metric of interest—stereotype *negativity—*by taking the historically-contextualized (Appendix) valence scores of these traits^19^. For example, in 1900, the group *Homeless* was most associated with traits including *helpless, heartless, lonely, disorderly,* and *thoughtless*, which had an average valence score of − 0.10 (corresponding to the 18th most negative group); in 1950, the group was associated with traits including *helpless, careless, inquisitive, impetuous,* and *cruel*, with an average valence score of − 0.11 (the 17th most negative). In this way, each of the 58 groups ends up with a timeseries of 11 valence scores (all decades from 1900 to 2000). Additionally, to have a measure of whether the stereotype was stable in latent semantic meaning, we transformed the top-associated traits into scores of stereotype *warmth* and *competence*^28,29^, a widely used typology of stereotype content. We again did so using historically-contextualized scores (Appendix) of each trait along these latent semantic dimensions. In summary, our analyses focus on the 58 timeseries (one for each group) of latent valence, warmth, and competence, in addition to changes in the top-associated traits themselves (i.e., the top-10 trait content).

**Figure 1:**
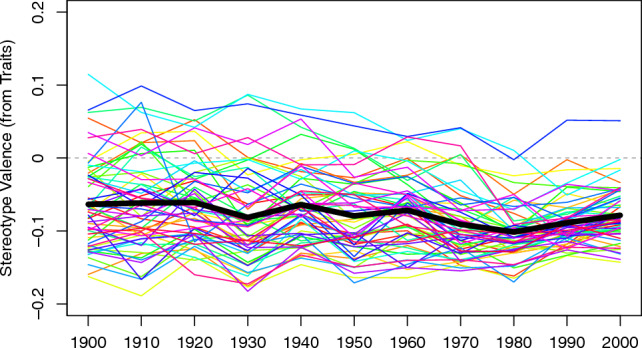
Trajectories of stereotype valence (positivity/negativity) towards 58 stigmatized groups. The dark black line indicates the aggregate (averaged) trajectory from raw values, showing stability in aggregate trends of stereotype negativity across 58 stigmatized groups over the past century. Individual colored lines show the individual group trajectories. Y-axis indicates the stereotype valence score (historically-contextualized valence scores averaging across the top 10 traits in each decade), with higher scores indicating more positive trait representations and lower scores indicating more negative trait representations. X-axis indicates the decade of the Google Books text.

For our first result, we inspect the average stereotype negativity aggregating across the 58 stigmatized groups over 100 years of English-language books. Bayesian mixed-effects models (*Methods*) showed an aggregate slope that was close to zero, *b* = − 0.0030, 95% credible interval (CI) [− 0.0042, − 0.0017] (Fig. 1), indicating only a slight movement towards more negative representations of stigmatized groups over the past century. Indeed, inference using the Region of Practical Equivalence (ROPE)^30^ showed 100% of the posterior estimates for the aggregate slope fell within a region that would be reasonably said to be a “null” effect. Thus, over 100 years of English-language text, negative stereotypes of stigmatized groups have remained, on aggregate, remarkably stable.

### Robustness checks

We ensured that central conclusions were robust to various methodological choices (Appendix). First, because the Google Books corpus changed over time in the proportion of non-fiction scientific texts^31^, we replicate all analyses with word embeddings trained on the smaller, genre-balanced (i.e., consistent balance of fiction and non-fiction texts over time) Corpus of Historical American English^32^. Although COHA is substantially smaller (< 1% the size of Google Books), we still find consistent conclusions with both corpora, ruling out concerns that the observed stability in stereotype negativity is due merely to changes in genre composition. Second, we ensured robustness across frequentist modeling approaches, finding identical conclusions regardless of model specifications.

Third, for a subset of groups we had scores on (1) how much the meaning of the group labels (e.g., changes in the meaning of *Gay*) had changed across time (known as semantic drift), (2) how many meanings the group labels had other than non-group related meanings (known as polysemy), and (3) the frequency of these labels. For groups with these available data, we computed an additional regression that directly controlled for drift, polysemy, and frequency of group labels, and the main conclusions of aggregate stability remained. Further, none of these three variables showed significant interactions with change, indicating that these covariates did not moderate the conclusions of how groups are changing. Fourth, because the current methods rely on choices of how to represent the social groups in question, we tested whether changing the lists of group labels altered the key results. Even when using only the four most central and frequent group labels to represent a sample of the groups, we again found aggregate stability.

## Study 2: mechanisms upholding aggregate negativity over 100 years of text

**Figure 2:**
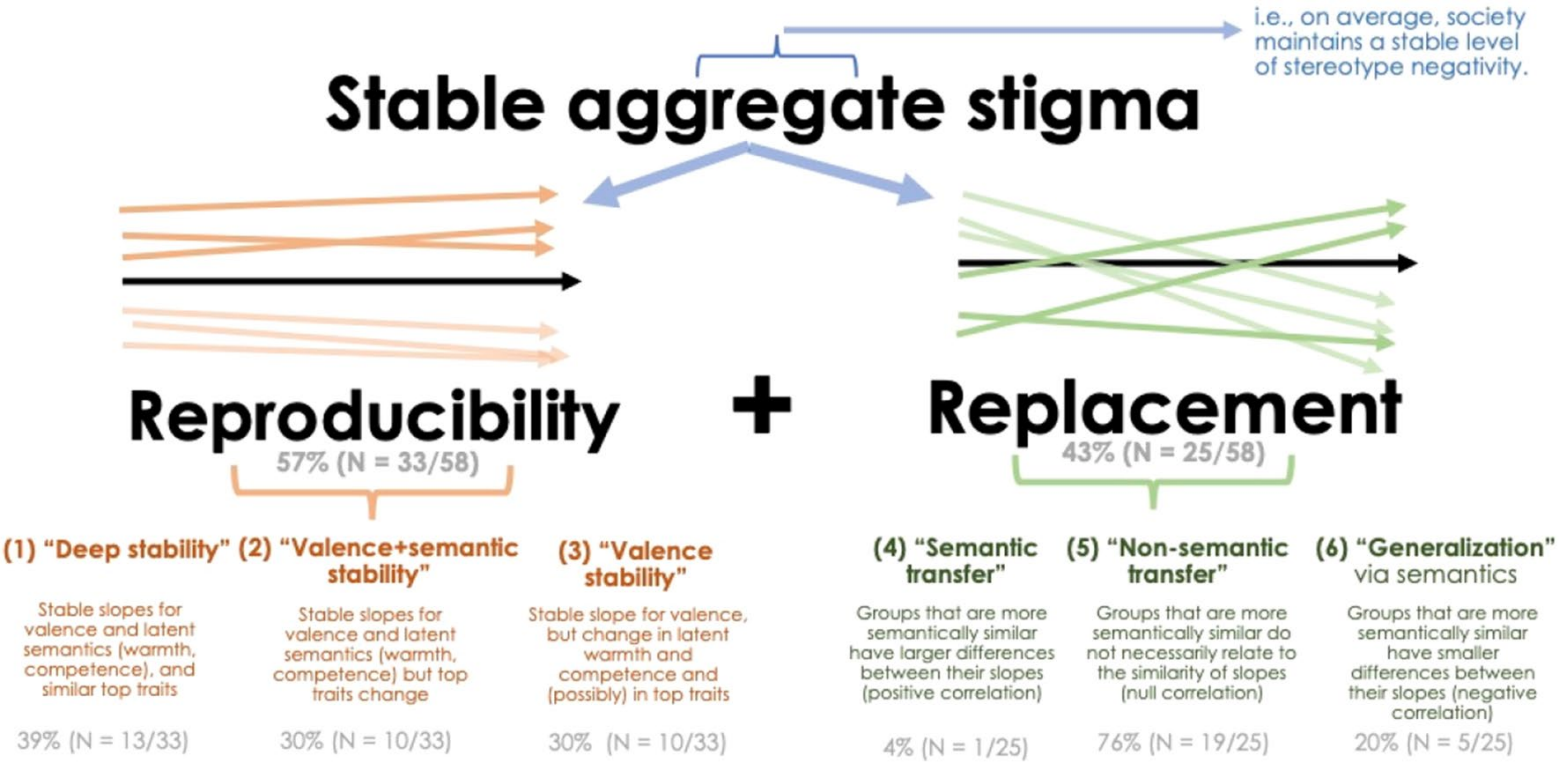
Visual overview of the Stigma Stability Framework. The framework proposes two complementary classes of mechanisms—replacement and reproducibility—to explain aggregate (averaged) persistence of negative stereotypes towards stigmatized groups at a societal level. The general mechanisms are, in turn, empirically enacted in six empirical patterns, as described in the figure. Gray numbers and percentages indicate the number of groups, in the current sample and with the current methods, that followed each empirical pattern.

Study 1 showed that aggregate stereotype negativity was relatively stable over 100 years of English-language book text, raising the question of what societal mechanisms might maintain such stigmatization. Here, we introduce and test the *Stigma Stability Framework* (Fig. 2) to propose two complementary mechanisms of reproducibility (within groups) and replacement (transfer across groups), each enacted in three empirical patterns.

### Reproducibility

We refer to the first mechanism as *reproducibility*, in which stereotype negativity is reproduced (repeated) towards a single target group. The idea of reproducibility emerges from the theory of stigma as a fundamental cause^26^, which posits that, if the underlying motivations to stigmatize (e.g., to dominate) have not been addressed, advantaged groups will continually reproduce stigma, often by developing new means to stigmatize the same group via interchangeable, mutually reinforcing mechanisms. For example, historical analyses show the changing means used to sustain stigmatization of Black people in the United States, moving from slavery to explicit forms of discrimination (e.g., Jim Crow laws) to more covert expressions, such as aversive^33^ and “laissez faire” racism^34^.

Empirically, reproducibility can be enacted through three patterns. First, a pattern we term “deep stability” occurs when a stereotype is repeated across time (e.g., a group is “lazy” in 1900 and “idle” in 2000), with the same underlying negativity, latent semantic meaning (i.e., warmth and competence dimension), and semantically-similar top associated traits (i.e., near synonyms with high cosine similarities). A second pattern, which we term “valence + semantic stability”, occurs when the same underlying negativity and semantic meaning is reproduced but new traits emerge; for example, a group is “lazy” in 1900 but “helpless” in 2000, with “helpless” being similar to “lazy” in average warmth and competence but not a direct semantic synonym as in the first pattern (i.e., they have lower cosine similarities), thereby reflecting change in the top trait associates. In a third pattern that we term “valence stability”, the same underlying negativity is reproduced, but the source of the negativity shifts as new semantic meanings become associated with the group (e.g., “lazy” in 1900 but “ugly” in 2000, where “lazy” and “ugly” are both negative but are different in latent warmth and competence).

### Replacement

The second complementary mechanism upholding aggregate stereotype negativity is stigma *replacement,* in which the negativity towards one group is transferred across group lines such that, on aggregate, patterns of change may “cancel out”. As one historical example, replacement is seen in increasing negativity towards Black Americans following the Great Migration into the Northern US in 1915–1930 that occurred alongside *decreasing* negativity towards European immigrant groups into those same areas^35^. That is, negativity historically held towards immigrants was transferred to more newly-arrived Black Americans. More broadly, the idea of replacement is also conceptually supported by the theory of stigma as a fundamental cause^26^: if underlying stigma motivations remain, but the permissibility of stigmatizing a given target changes (e.g., it is no longer permissible to stigmatize immigrant groups), then advantaged groups may seek a new target for their negativity (e.g., Black Americans).

Replacement, understood as the transfer or generalization of negativity, can similarly follow three empirical patterns. First, replacement could occur by transferring stereotype negativity across groups that share some semantic relationship, essentially in a hydraulic manner. For example, lessening negativity towards *Asexual* people may be transferred towards a group that shares similar semantic meanings of warmth and competence (i.e., is represented close in semantic space)*,* such that a group like *Infertile* experiences a corresponding strengthening in negativity. In this case, groups that are more semantically similar in the 1900s would have very different (and, perhaps in some cases, even opposing) slopes from 1900 to 2000, resulting in a negative correlation between semantic similarity between groups and similarity in their change.

Second, the transferal of negativity could occur through other, non-semantic processes. Empirically, this would be observed if the semantic similarity between groups did not significantly predict the similarity of negativity slopes across groups. Indeed, the above example of transferring prejudice between immigrant groups and Black Americans^35^ is less about shared semantics than it is other shared characteristics, such as geographic location. Further explanations for why negativity is transferred between groups could include the relative prevalence of the groups (e.g., when a group switches from the second to the first most prevalent minority group in society it could “acquire” the brunt of prejudice^25^) or the shared functions of the groups (e.g., both satisfy the need for exploitation^36^).

Finally, also within the general umbrella of replacement is a pattern that we term “generalization”, which is the idea that some semantically-related groups may experience similar patterns of lessening negativity; in short, a pattern of change in one group “generalizes” to a similar second group, such that there would be a negative correlation between the semantic similarity between groups and their differences in change. To be clear, this pattern is not a hydraulic relationship (i.e., one group lessens, another group strengthens) like the other two empirical patterns of replacement, and thus it is not strictly a means of maintaining aggregate stable negativity. In fact, observing “generalization” would result in an aggregate *change* in the societal-level of negativity because multiple groups are changing in similar ways and similar directions. Nevertheless, we include this last empirical pattern under the umbrella of a “replacement” mechanism, because it conceptually also involves a transfer or generalization of negativity across groups.

## Results

Study 2 used the same data and general methods as Study 1 to provide initial empirical tests of the prevalence of mechanisms in the Stigma Stability Framework, looking across all 58 groups and 100 years of English-language book text. A group is classified as showing *reproducibility* if the random slope estimates from the Bayesian regression model are null (i.e., the Highest Density Interval includes zero). Conversely, a group is classified as showing *replacement* if the random slopes are not null, since replacement requires that the target group be changing in stereotype negativity for some transfer to occur.

### Overall prevalence of mechanisms

Using these criteria, we found that over half of the individual group-level slopes (33/58 groups; or 57%; Table 1) revealed little meaningful change, a result consistent with the reproducibility mechanism. The remaining groups showing change (25/58; or 43%) suggest replacement (transfer) of stereotype negativity.

**Table 1:** Estimated change in latent valence and identified top traits for 58 stigmatized groups across 100 years of book text.

Group	Mechclass	Change in latent valence *b* [95% HDI]	Top 10 traits	
			1900	2000
Bipolar	1	− 0.0019 [− 0.0055, 0.0015]	Depressed, nervous, despondent, melancholy, intellectual, irritable, intense, moody, restless, emotional	Depressed, compulsive, antisocial, severe, emotional, patient, unstable, persistent, temperamental, aggressive
Blind	1	0.0005 [− 0.0028, 0.0038]	Stupid, dull, superstitious, foolish, careless, relaxed, melancholy, ignorant, irritable, conceited	Lustful, dull, sloppy, gullible, listless, spiteful, suave, scornful, stingy, glum
Depressed	1	0.0009 [− 0.0027, 0.0045]	Melancholy, gloomy, depressed, despondent, listless, lonely, moody, fearful, irritable, helpless	Depressed, gloomy, angry, fearful, melancholy, listless, lonely, anxious, dull, helpless
Gang	1	0.0010 [− 0.0025, 0.0045]	Noisy, disorderly, unruly, ruthless, fanatical, reckless, greedy, spiteful, dishonest, hostile	Spiteful, disrespectful, bossy, glum, inconsiderate, stingy, lustful, sloppy, suave, opinionated
Heartattack	1	− 0.0023 [− 0.0058, 0.0012]	Nervous, sympathetic, superficial, irritable, severe, perceptive, patient, tense, excitable, persistent	Severe, patient, spontaneous, nervous, unstable, sympathetic, persistent, compulsive, moderate, abrupt
Homeless	1	0.0002 [− 0.0032, 0.0038]	Helpless, heartless, lonely, disorderly, thoughtless, conceited, reckless, adventurous, unruly, greedy	Spiteful, sloppy, opinionated, despondent, suave, insolent, bossy, headstrong, rebellious, disrespectful
Mute	1	− 0.0015 [− 0.0049, 0.0019]	Silent, despondent, helpless, meek, submissive, timid, listless, melancholy, lifeless, dull	Silent, listless, dull, easygoing, suave, fussy, argumentative, spiteful, amiable, disrespectful
Prostitute	1	− 0.0014 [− 0.0048, 0.0020]	Conceited, headstrong, immoral, moody, heartless, enterprising, crafty, thoughtless, resentful, disorderly	Stingy, lustful, bossy, sociable, spiteful, suave, opinionated, disrespectful, indiscreet, envious
Ret*	1	− 0.0029 [− 0.0065, 0.0005]	Stupid, cowardly, unsympathetic, ignorant, mischievous, intellectual, impressionable, timid, sluggish, immoral	Stupid, opinionated, fussy, easygoing, temperamental, immature, abusive, spiteful, listless, compulsive
Scarred	1	0.0002 [− 0.0033, 0.0036]	Helpless, lifeless, cruel, weak, heartless, despondent, spiteful, tender, clumsy, irritable	Dull, superstitious, clumsy, careless, insolent, rude, helpless, listless, spiteful, easygoing
Short	1	− 0.0015 [− 0.0050, 0.0020]	Intolerant, arrogant, thoughtless, shy, timid, fickle, conceited, mischievous, touchy, fearful	Spiteful, listless, suave, sloppy, headstrong, envious, scornful, gloomy, lustful, opinionated
Stroke	1	− 0.0020 [− 0.0054, 0.0014]	Nervous, severe, patient, irritable, impressionable, relaxed, perceptive, intense, passive, cold	Severe, nervous, patient, spontaneous, compulsive, rash, irritable, persistent, progressive, depressed
Unattractive	1	0.0005 [− 0.0031, 0.0041]	Clumsy, despondent, rude, vulgar, disagreeable, stupid, gloomy, coarse, pompous, shy	Spiteful, listless, easygoing, stupid, opinionated, inept, gloomy, suave, greedy, headstrong
Abortion	2	− 0.0023 [− 0.0060, 0.0012]	Impressionable, spontaneous, immature, artificial, inventive, rash, patient, perceptive, rebellious, rational	Cruel, immoral, prudent, impolite, incompetent, spontaneous, considerate, pompous, fearful, antisocial
Alcoholic	2	− 0.0023 [− 0.0059, 0.0012]	Cold, impulsive, sensual, irritable, weak, haphazard, nervous, sober, sluggish, greedy	Compulsive, antisocial, irritable, reckless, disorderly, abusive, negligent, temperamental, emotional, severe
Black	2	0.0005 [− 0.0030, 0.0042]	Fanatical, spiteful, cultured, greedy, belligerent, coarse, reckless, helpless, honest, irresponsible	Bossy, squeamish, talented, thoughtless, insolent, aloof, belligerent, respectable, sloppy, disrespectful
Cancer	2	− 0.0018 [− 0.0051, 0.0016]	Superficial, patient, nervous, irritable, progressive, severe, spontaneous, perceptive, intense, passive	Cultured, spontaneous, patient, nervous, superficial, immature, severe, inactive, blunt, soft
Divorced	2	− 0.0028 [− 0.0064, 0.0008]	Detached, unruly, independent, immature, dependent, unkind, helpless, dominant, respectable, aloof	Detached, abusive, incompetent, dependent, unkind, resentful, respectable, jealous, mature, fussy
Herpes	2	− 0.0027 [− 0.0061, 0.0008]	Nervous, bright, impressionable, irritable, severe, bland, patient, cold, coarse, erratic	Severe, antisocial, cultured, nervous, persistent, compulsive, unconventional, patient, rash, spontaneous
Molestor	2	0.0001 [− 0.0035, 0.0036]	Lenient, obnoxious, vindictive, disorderly, cruel, severe, unjust, unfair, ruthless, reasonable	Disorderly, antisocial, discriminating, abusive, inept, aggressive, manipulative, temperamental, impulsive, compulsive
Muslim	2	− 0.0005 [− 0.0040, 0.0033]	Hostile, fanatical, ruthless, arrogant, rebellious, daring, insolent, stubborn, belligerent, irresponsible	Religious, bossy, belligerent, aloof, suave, opinionated, inconsiderate, stingy, benevolent, superstitious
Schizophrenic	2	− 0.0030 [− 0.0064, 0.0004]	Sophisticated, immature, impressionable, irritable, squeamish, perceptive, nervous, feminine, spiteful, severe	Compulsive, antisocial, temperamental, depressed, patient, severe, emotional, spontaneous, immature, spiteful
Wheelchair	2	− 0.0007 [− 0.0043, 0.0028]	Helpless, patient, irritable, weak, depressed, inactive, severe, nervous, inefficient, unsympathetic	Helpless, incompetent, abusive, patient, emotional, severe, negligent, irritable, depressed, immature
Atheist	3	0.0026 [− 0.0009, 0.0062]	Fanatical, intolerant, cowardly, vulgar, arrogant, obstinate, rebellious, superstitious, fickle, cruel	Spiteful, bossy, stingy, opinionated, argumentative, disrespectful, fickle, headstrong, sloppy, insolent
Criminal	3	0.0025 [− 0.0011, 0.0061]	Disorderly, immoral, cruel, obnoxious, dishonest, vindictive, heartless, cowardly, negligent, ruthless	Disorderly, incompetent, negligent, dishonest, reckless, prejudiced, unethical, cruel, suspicious, conscientious
Deaf	3	0.0006 [− 0.0030, 0.0042]	Dull, articulate, depressed, harsh, irritable, despondent, resentful, melancholy, weak, noisy	Severe, spontaneous, depressed, abusive, argumentative, thoughtless, listless, temperamental, emotional, spiteful
Disabled	3	0.0023 [− 0.0011, 0.0058]	Helpless, cowardly, inefficient, incompetent, negligent, irritable, resentful, inactive, weak, cruel	Abusive, helpless, incompetent, negligent, inept, opinionated, thoughtless, fussy, argumentative, discriminating
Infertile	3	0.0014 [− 0.0020, 0.0049]	Lonely, helpless, envious, heartless, arrogant, cruel, mischievous, fickle, lifeless, harsh	Inept, abusive, listless, immature, unruly, vindictive, incompetent, irresponsible, easygoing, helpless
Middleeastern	3	0.0024 [− 0.0011, 0.0062]	Hostile, ruthless, cruel, fanatical, rebellious, insolent, belligerent, irresponsible, crafty, ignorant	Belligerent, diplomatic, bossy, hostile, indecisive, despondent, fanatical, spiteful, inconsiderate, loyal
Molested	3	0.0018 [− 0.0016, 0.0053]	Ruthless, worried, insolent, disorderly, cruel, prejudiced, vindictive, dishonest, fanatical, hostile	Jealous, angry, abusive, disagreeable, suspicious, prejudiced, impetuous, shrewd, unethical, incompetent
Polygamous	3	0.0002 [− 0.0031, 0.0035]	Cooperative, aggressive, immoral, dominant, illogical, optimistic, religious, spontaneous, superstitious, unfriendly	Stingy, spiteful, suave, bossy, disrespectful, temperamental, inconsiderate, glum, gullible, opinionated
Poor	3	0.0012 [− 0.0024, 0.0049]	Helpless, heartless, ignorant, weak, cowardly, conceited, lazy, stupid, envious, cruel	Helpless, stupid, ignorant, easygoing, lazy, hardworking, envious, greedy, opinionated, rebellious
Unemployed	3	− 0.0009 [− 0.0043, 0.0025]	Inactive, listless, lazy, frivolous, restless, noisy, helpless, inefficient, silent, aloof	Inactive, lazy, ignorant, helpless, depressed, inefficient, weak, restless, immature, listless
Asexual	4	− 0.0121 [− 0.0160, − 0.0085]	Strict, conventional, meek, submissive, feminine, conscientious, dignified, modest, religious, compassionate	Opinionated, spiteful, suave, sloppy, squeamish, inconsiderate, easygoing, argumentative, disrespectful, fickle
Aboriginal	5	− 0.0046 [− 0.0081, − 0.0011]	Hostile, crafty, adventurous, ruthless, fanatical, rebellious, rude, belligerent, devious, fickle	Bossy, sloppy, stingy, spiteful, carefree, devious, squeamish, touchy, disrespectful, egotistical
Asian	5	− 0.0043 [− 0.0079, − 0.0006]	Crafty, dominant, belligerent, hostile, traditional, diplomatic, irresponsible, cultured, adventurous, cooperative	Bossy, belligerent, diplomatic, religious, traditional, peaceful, social, unconventional, concise, devious
Christian	5	− 0.0055 [− 0.0091, − 0.0018]	Religious, fanatical, tolerant, dominant, spiritual, hostile, cultured, intolerant, ethical, loyal	Bossy, religious, conservative, spiritual, benevolent, loyal, philosophical, argumentative, stingy, moral
Dealer	5	− 0.0071 [− 0.0108, − 0.0036]	Talkative, smart, haphazard, shrewd, skillful, crafty, sly, cunning, dishonest, possessive	Compulsive, gullible, unethical, disorderly, nonchalant, unscrupulous, inept, antisocial, fickle, negligent
Fat	5	− 0.0056 [− 0.0091, − 0.0020]	Tough, soft, strong, weak, clumsy, coarse, jolly, earthy, neat, sluggish	Easygoing, coarse, moderate, soft, immature, listless, lazy, fussy, bright, witty
Gay	5	− 0.0095 [− 0.0132, − 0.0060]	Spiteful, squeamish, jolly, curious, humorous, pompous, sentimental, bashful, touchy, clumsy	Lustful, bossy, spiteful, opinionated, argumentative, suave, easygoing, temperamental, disrespectful, fussy
HIV	5	− 0.0132 [− 0.0170, − 0.0095]	Spiritual, ethical, indirect, artistic, efficient, intellectual, rational, artificial, helpful, inventive	Severe, patient, antisocial, persistent, nervous, temperamental, inactive, abusive, capable, compulsive
Indian	5	− 0.0043 [− 0.0078, − 0.0008]	Ignorant, superstitious, traditional, crafty, hostile, abusive, religious, intelligent, friendly, polite	Bossy, stingy, belligerent, squeamish, religious, sloppy, spiteful, inconsiderate, glum, concise
Jewish	5	− 0.0044 [− 0.0079, − 0.0008]	Religious, traditional, spiritual, ethical, fanatical, materialistic, cultured, dominant, intolerant, tolerant	Religious, bossy, spiritual, benevolent, conservative, superstitious, uncompromising, loyal, traditional, hostile
Laborer	5	− 0.0117 [− 0.0153, − 0.0081]	Honest, skillful, intelligent, diligent, respectable, talented, jolly, ingenious, cooperative, practical	Respectable, suave, opinionated, diligent, benevolent, ignorant, spiteful, gullible, talented, bossy
Latino	5	− 0.0052 [− 0.0087, − 0.0017]	Belligerent, hostile, diplomatic, adventurous, loyal, rebellious, friendly, indecisive, crafty, uncompromising	Belligerent, bossy, diplomatic, social, squeamish, opinionated, bland, spiteful, disrespectful, devious
Old	5	− 0.0080 [− 0.0116, − 0.0041]	Respectable, impressionable, affectionate, discreet, helpless, amiable, thoughtful, mature, proud, considerate	Affectionate, respectable, proud, talented, jealous, amiable, charming, compassionate, fussy, submissive
Paroled	5	− 0.0044 [− 0.0079, − 0.0011]	Evasive, rash, flattering, satisfied, unfair, unjust, negligent, responsible, indiscreet, belligerent	Incompetent, dishonest, negligent, unscrupulous, cruel, competent, indiscreet, spiteful, compulsive, impartial
Pierced	5	− 0.0060 [− 0.0094, − 0.0026]	Clumsy, blunt, lifeless, tough, spiteful, bright, deep, coarse, soft, expressive	Suave, glum, sloppy, spiteful, easygoing, stingy, listless, squeamish, bossy, untidy
Psoriasis	5	− 0.0039 [− 0.0075, − 0.0005]	Nervous, irritable, severe, soft, superficial, patient, bright, tense, relaxed, harsh	Rash, severe, compulsive, nervous, excitable, irritable, persistent, cultured, spontaneous, touchy
Server	5	− 0.0091 [− 0.0128, − 0.0055]	Jolly, touchy, smart, haphazard, tidy, fickle, neat, merry, lazy, charming	Suave, easygoing, fussy, cheerful, jolly, noisy, glum, merry, disrespectful, sly
Std	5	− 0.0042 [− 0.0076, − 0.0008]	Nervous, perceptive, impressionable, immature, helpful, severe, competitive, patient, artificial, direct	Antisocial, patient, cultured, severe, nervous, persistent, compulsive, unconventional, inactive, rash
Transgender	5	− 0.0091 [− 0.0128, − 0.0056]	Methodical, philosophical, original, analytical, devious, outgoing, argumentative, constructive, ethical, impersonal	Bossy, argumentative, squeamish, egotistical, suave, gullible, sloppy, indiscreet, spiteful, philosophical
Uneducated	5	− 0.0048 [− 0.0084, − 0.0014]	Ignorant, vulgar, stupid, conceited, intelligent, careless, submissive, inquisitive, indifferent, thoughtless	Ignorant, glum, opinionated, suave, bossy, easygoing, spiteful, intelligent, stingy, inept
Diabetic	6	− 0.0044 [− 0.0079, − 0.0009]	Severe, patient, sophisticated, nervous, analytical, impressionable, progressive, irritable, bright, immature	Severe, temperamental, compulsive, spontaneous, dependent, patient, immature, nervous, depressed, progressive
Drugaddict	6	− 0.0042 [− 0.0077, − 0.0007]	Earthy, crude, discriminating, weak, disagreeable, coarse, bitter, cold, fickle, sluggish	Compulsive, antisocial, abusive, inept, temperamental, rebellious, spiteful, irritable, heartless, opinionated
Immigrant	6	− 0.0058 [− 0.0093, − 0.0024]	Hostile, unfriendly, ignorant, adventurous, dominant, enterprising, greedy, irresponsible, rebellious, disorderly	Abusive, hostile, belligerent, aloof, heartless, ruthless, ignorant, prejudiced, casual, headstrong
Multiracial	6	− 0.0053 [− 0.0089, − 0.0016]	Tidy, spiteful, greedy, spirited, shy, bashful, immature, benevolent, indiscreet, fearless	Social, bossy, argumentative, disrespectful, benevolent, temperamental, sloppy, feminine, religious, insolent
Smoker	6	− 0.0060 [− 0.0096, − 0.0026]	Sly, listless, sociable, coarse, bland, neat, scornful, jolly, sarcastic, lazy	Compulsive, antisocial, soft, nonchalant, persistent, listless, irritable, refined, spontaneous, disagreeable

“Mech. class” indicates the class of mechanism for each group, with codes as follows (see also Fig. 2): “1” = reproducibility via “deep stability,” with all metrics showing stability over time; “2” = reproducibility via “valence + semantic stability,” despite change in the top traits; “3” = reproducibility via “valence stability,” despite changing semantic sources of the valence; “4” = replacement via “shared semantics”; “5” = replacement or transfer via other, “non-semantic” means; “6” = the only empirical pattern that might produce change, by producing “generalization” via shared semantics, where a change in one group cascades to similar changes among semantically-related groups. Estimated change in latent valence are the group-level random slopes, from the Bayesian mixed-effects model predicting valence from the fixed effect of time. 95% HDI = 95% Highest Density Interval. Top traits are the top-10 traits ranked as the most associated with the group target (i.e., have the highest average cosine similarities), within the listed example years.

### Empirical patterns of stability through reproducibility

Starting with the 33 groups indicating reproducibility, we find evidence for all three proposed patterns, each occurring in approximately equal proportion. The first pattern (“deep stability”) is descriptively the most common, observed in 13/33 groups (39%). For example, negative stereotypes of the group *Mute* had top traits including [*silent, listless, dull*] in 1900 and 2000, with both timepoints reflecting near-identical negative representations (with traits that had high cosine similarities) and reflecting the same latent stereotype meanings of coldness and incompetence across time.

A second set of groups, 10/33 (30%), followed the “valence + semantic stability” pattern in which the actual top-associated traits turned over across time (i.e., traits had low cosine similarities) but latent valence and warmth and/or competence were stable. The negative stereotypes towards *Black* illustrate this empirical pattern: top traits in 1900 included [*coarse, reckless, irresponsible, helpless, honest*] but in 2000 included [*sloppy, belligerent*, *thoughtless*, and *respectable*]. Although the traits themselves changed, the negativity was reproduced via stable latent sources (i.e., the average latent warmth was stable, *b* = 0.0024 [− 0.0001, 0.0050], as was average competence *b* = − 0.0002 [− 0.0031, 0.0026]).

The final set of groups, 10/33 (30%), followed the “valence stability” pattern, wherein latent valence was stable and reproduced across time, but the source of that valence varied (i.e., the latent semantics of warmth and competence shifted, possibly also with changes in the top traits). For example, stereotypes of *Criminal* were persistent in negative valence (*b* = 0.0025 [− 0.0011, 0.0061]) but the traits also showed an increase in latent warmth (*b* = 0.0036 [0.0009, 0.0063], see Appendix. That is, although the new top-associated traits were (relatively) warmer (e.g., no longer *harsh* and *cruel* but now *inept* and *immature*), they continued to reproduce negative valence through other meanings, such as by increasing in negative competence, negative morality, and assertiveness^37^. In sum, for these latter groups we find that, when a new trait does emerge, it likely brings new meaning along latent axes of warmth/competence or some other dimension, but always reproducing the underlying negative valence.

### Empirical patterns of stability through replacement

Twenty-five groups (43%) changed meaningfully in stereotype negativity, prompting the next investigation on which empirical patterns of replacement they follow (Fig. 2). The first possibility is a “transfer” of stigma via shared semantics, in which a strengthening negativity towards one target group corresponds to diminishing negativity in secondary groups that are semantically related. Such a pattern was notably rare in the groups we examined. Indeed, using our current empirical operationalization (*Methods, Appendix*), only one group, *Asexual*, suggested transfer via shared semantics (i.e., warmth/competence) with other groups: *Asexual* showed a strong negative slope, *b* = − 0.012, while semantically-similar groups including *Infertile* (*b* = 0.0014) and *Atheist* (*b* = 0.0026) had slopes that were null but trended towards more positivity over time. In short, the initial tests appear to suggest transfer via shared semantics is a relatively rare mechanism in historical patterns of stigma negativity, although it could be observed more widely for other groups using different empirical criteria.

In contrast, most of the changing groups (19/25, or 76%) suggested other processes of transferring negativity that were not predicted by simple semantic relationships. For instance, the increasing negativity towards the group target *Aboriginal* did not correspond to lessened negativity towards semantically-related groups of *Indian* or *Middle-eastern*, suggesting that many changing groups may be sharing/transferring negativity through processes not reducible to shared semantics.

Finally, we found that a handful of the changing groups (5/25, or 20%) showed “generalization” of negativity, in which semantically-similar groups are changing in similar ways (e.g., similar strengthening in negativity). For instance, increasing negativity towards *Smoker* (*b* = − 0.0060) was similar and shared across semantically-similar groups including *Alcoholic* (*b* = − 0.0023). Such a finding could help explain why the overall, aggregate trend showed a slight movement towards more negative representations in general. We nevertheless emphasize that this empirical pattern of generalization is uncommon (only 5 groups out of the possible 58), thereby underscoring that mechanisms prompting widespread change in societal negativity are rare in the current set of stigmatized groups.

## General discussion

Using 100 years of English-language book text and the largest sample of negatively stigmatized groups studied via NLP methods to date, the current research contributes new understanding to the persistence of aggregate negativity in social group stereotypes. Study 1 shows that, over the past 100 years, societies have maintained a relatively stable level of stereotype negativity, as revealed from the aggregate trend across 58 stigmatized groups. A key contribution of the current work is going beyond this aggregate persistence to also consider what societal mechanisms may uphold such negativity. Study 2 provided a first attempt at conceptualizing and empirically testing a novel theoretical framework for addressing this question. We propose two overarching classes of mechanisms—reproducibility of negativity towards individual group targets, and replacement (or transfer) of negativity across group lines—as a framework to understand how stereotype negativity persists on aggregate. The initial empirical tests of this framework suggest three key take-aways.

First, the reproducibility mechanism is relatively more prevalent than replacement, with 57% of groups showing individual stable slopes, suggesting that negativity itself is reproduced towards individual group targets. Within these stable groups, approximately one-third showed “deep stability” (i.e., all metrics we investigated were stable), as in the case of several disability-related stigmas. The remaining two-thirds of stable groups showed patterns of reproducibility that suggested shifting sources of negativity. For instance, for groups such as *Alcoholic* or *Black*, the top-associated traits might have shifted over time, but the underlying latent valence was always the same general level of negativity. Such dynamic reproducibility suggests that society may be inventing new means (e.g., new words or new meanings) to repeatedly stigmatize the same groups across time^38^.

At the same time, a handful of groups did show some meaningful change in stereotype negativity, underscoring that change for some groups is possible, if far from assured. Such change in negativity suggests the operation of a complementary *replacement* mechanism, in which negativity is transferred across group lines. Notably, however, we found little evidence that the transfer of stigma was falling along predictable lines of semantically-similar groups (e.g., there was no evidence of a transfer between *Gay* and *Transgender*^39^). Instead, the data suggest that transfer of stigma is more likely to occur through means other than simply semantic relations. These findings set the stage for future research to identify non-semantic replacement mechanisms, such as groups that appear in the same geographic locations^35^, that fulfill the same function^36^, or that switch their relative ranking in terms of numerical prevalence^25^.

Finally, for a small handful of changing groups, we found that increasing negativity towards one target group appeared to cascade through semantically-related groups, an empirical pattern that could help explain the slight aggregate trend towards increasingly negative representations over time. That is, while the current work focuses on the mechanisms upholding stigma stability, we also show the utility of the current methods for uncovering means by which society may, in the future, show aggregate change in stigmatization. Although such generalization of stigma is obviously concerning in the case of increasing *negativity*, it could be possible that, for other groups not studied here (e.g., groups that are not as ubiquitously stigmatized), generalization mechanisms could operate to cascade *positivity* throughout the network (e.g., as in the “secondary transfer effects” of intergroup contact^40^).

Of course, there are limitations to using text analysis for social science inquiries. For instance, when it comes to the words used to operationalize groups, factors such as semantic drift, polysemy, and frequency^14^ can confound inferences. In the Appendix, we show that the primary conclusions are not altered after controlling for the drift, polysemy, or frequency of group labels, or after using shorter lists of only four central group words. Additionally, when it comes to the underlying text, the current study focused on the (limited) Google Books English corpus^31^. Although conclusions were robust in a complementary book text source, variation is likely to arise in different media sources or languages. For instance, stigma may be more persistent in some societies than others, such as those with stronger collective norms that require more conformity^41^. We look forward to testing such questions following continued innovations in natural language processing and the availability of archived text data across cultures, geographic locations, and diverse languages.

Finally, the current work was limited in focusing on only one dimension of stigma—negativity in stereotypes—leaving open the question of how other aspects of stigma, such as the initial act of labeling or behavioral dimensions of discrimination^1^, might persist or change over time. Although labeling and behavior are more difficult to address using historical language, researchers may successfully merge the current data with other indicators of stigmatization such as the persistence of discrimination in audit experiments^42,43^ or human attitude data^22,44^ to better understand the persistence and change of interacting components of stigma^6^.

## Conclusion

The results reported here fall between the hopes of optimists that we might gradually increase in positivity towards all groups^22^ and the fears of pessimists that society will continue to grow in hostility and negativity^24^. Instead, the current data seem to suggest a stasis, in which the aggregate negativity of today is not so different from that of the past. Most critical, by expanding beyond traditional social science methods to consider stereotype negativity towards a large, diverse set of stigmatized groups across an unprecedented timespan of 100 years of books, we can also newly observe *how* stigmatization persists in society. Our hope is that introducing the Stigma Stability Framework, alongside a methodological toolkit to test its predictions, will provide a clearer path to explore the mechanisms (specifically, reproducibility and replacement) upholding persistent negativity. Only by understanding the pernicious ways that stigma endures both within and across groups can we, as researchers and societal actors, be equipped to durably reduce the multifaceted processes of stigmatization.

## Methods

### Text data sources

We used word embeddings trained using the *word2vec* algorithm (a neural network method to compute vector representations of word meaning^45^) on book text obtained from Google Books and the Corpus of Historical American English (COHA) text data ^14^. Standard hyperparameters were used (e.g., 300-dimensions, a context window size of 4 words on either side of the target training word), and only words appearing at least 500 times were included in training. The entirety of the Google Books corpus (across 200 years available, from 1800 to 2000) consists of ~ 850 billion tokens and 500 million books, while the COHA corpus is much smaller, consisting of ~ 410 million tokens, but it is balanced in the composition of text genres across history (equivalent balance of fiction and non-fiction texts).

### Selecting and representing stigmatized groups in text

A study of whether stigma is stable or changing in society requires the best approximation of a large, diverse set of stigmatized groups. To that end, we selected an established list of 93 stigmatized identities, characteristics, and statuses^46^. Because we use single word embeddings, a subset of these 93 groups were indistinguishable from one another with the current methods. Thus, we collapsed these into a single identity—for example, both “symptomatic” (e.g., *bipolar symptomatic*) and “remitted” identities (e.g., *bipolar remitted*) were combined, as were various forms of cancer (e.g., *breast cancer current, breast cancer remitted, colorectal cancer current*, and so on). We recognize this as a limitation of the current methods, since these groups do indeed differ in how they are perceived in society as well as in their social, health, and economic consequences.

To identify group stereotypes in text, we need to use multiple terms to represent a single group and thereby ensure that the representation of a group triangulates on the group-specific meaning rather than some other polysemous meaning of a single term (e.g., “Alien” alone could refer to aliens from outer space, rather than to the intended meaning of a non-citizen or immigrant). Thus, for each of the stigmatized groups, we generated lists of single word synonyms using both historical and contemporary thesauruses (e.g., *Oxford Historical Thesaurus, Thesaurus.com*). Table S1 in the Appendix lists the chosen synonyms for each group. Using only the uniquely distinguishable groups, and those groups that could be represented in a list of single word synonyms available across all decades of text, ultimately left us with a final list of 58 stigmatized groups (Table 1).

### Extracting stereotype content and valence

To compute stereotype valence (positivity/negativity), we begin by extracting the stereotype content (top-ten traits associated with each group). Using a list of 414 traits, all available traits in the corpus of text^27^, we computed the average cosine similarity between a given target trait (e.g., “untrustworthy”) and a group representation (e.g., *Dealer*), by averaging across the pairwise cosine similarities between the trait and all group synonyms (e.g., “untrustworthy”-*dealer,* “untrustworthy”-*peddler,* “untrustworthy”-*narcotic,* “untrustworthy”-*supplier,* and so on). Then, all traits were ranked according to how strongly associated they were with the group, and the top-ten traits were used as the stereotype content for that group in a given decade. Additional details are provided in the Appendix.

After identifying the top-10 trait associates for each group in each decade, we replaced the traits with their corresponding valence rating that was contextualized to that specific decade. Specifically, rather than assume that a single rating of valence (e.g., from valence rating norms) was applicable across 100 years, we allowed the valence of traits to vary across time. To do so, we first created lists of 25 words that strongly (and stably) signaled positivity/negativity, drawn from the lists used for the Implicit Association Test and the Word Embeddings Association Test. Then, we took each of the traits and looked at its relative cosine similarity to these positive and negative words within each decade of text. We used these historically-contextualized valence scores of each trait within a decade of text and took the average across all the top-10 traits within a decade. For example, imagine the top ranked traits for *Aboriginal* include [hostile, rebellious, adventurous, superstitious]. The corresponding historically-contextualized valence ratings for each of these traits in 1900 are [− 0.13, − 0.18, 0.05, − 0.19] and in 2000 are [− 0.14, − 0.11, − 0.02, − 0.15]. Taking the average across these traits returns an average valence for *Aboriginal* of − 0.11 in 1900 and − 0.11 in 2000. We repeat this computation for all 11 decades (1900–2000) resulting in an 11-decade long timeseries of average historically-contextualized valence scores for each stigmatized group.

We followed a similar process to create the timeseries for the average historically-contextualized latent warmth and competence scores for each stigmatized group. We use a set of “anchor” words (Appendix) from automated dictionaries that represent poles of warmth/coldness and competence/incompetence ^28,37^, and score each of the 414 traits in terms of its relative warmth/coldness or competence/incompetence within each decade of text. Then, for each decade, we replace the top-10 traits with its warmth (competence) score and calculate the average warmth (competence) score for the 11 decades.

### Modeling aggregate and individual group persistence or change

We fit a Bayesian mixed effects model (i.e., allowing each group to start at a different valence and change at a different rate) to the data frame of the 58 timeseries trajectories (for valence and, separately, for latent warmth and competence). Model specifications used a uniform prior, random intercepts and random slopes for each group, and all other default parameters and model specifications (e.g., 2000 iterations, 4 chains) using *brms* (package version 2.17.0) in R^47^. For inference, we focus on the region of practical equivalence or ROPE^30^, which is a pre-specified range of values that would reasonably be seen as indicating a “null effect.” Following convention, we set the ROPE to + /− 0.1*SD_y_ (with more conservative thresholds of 0.05*SD_y_ tested for robustness) and compute the percentage of the model’s posterior inside the ROPE to quantify evidence in favor of the null for the fixed effect of time (the overall population effect).

Additionally, we use the random effects of the individual group slope estimates to identify those groups that have meaningfully changed (and thus suggest replacement) or remained stable (suggesting reproducibility). For random effects, we use the 95% Highest Density Intervals (HDIs) and determine those changing groups as any group with an HDI not including zero in the estimated random effect; stable groups are any group with an HDI that includes zero.

### Supplementary Information

Supplementary Information.

## Data Availability

All data and R code are made publicly available at the project’s OSF page: https://osf.io/8p7s5/.
