# Supplementary material for: Mechanisms upholding the persistence of stigma across 100 years of historical text
Source: Sci Rep. 2024 May 14;14:11069. doi: 10.1038/s41598-024-61044-z (PMC11094178; doi:10.1038/s41598-024-61044-z)
Supplement: Supplementary file 1 — Supplementary Information. [file 41598_2024_61044_MOESM1_ESM.docx]

***SI Appendix* for:**

Mechanisms Upholding the Persistence of Stigma across 100 Years of Historical Text

All data and analyses described in this document are available at OSF: <https://osf.io/8p7s5/>

**[temporary view-only link for peer review:** <https://osf.io/8p7s5/?view_only=f009840a102a4e198ea54c0904e9b136>]

Table of Contents

[Additional details on text data sources 3](#_Toc165022116)

[Stigma selection process 4](#_Toc165022117)

[Computing stereotype content and latent valence, warmth, and competence 8](#_Toc165022118)

[Main models: Bayesian linear mixed effects models 12](#_Toc165022119)

[Additional details on mechanism of replacement: Correlations across groups 17](#_Toc165022120)

[Replication of main analyses with frequentist models 23](#_Toc165022121)

[Replication with COHA dataset 24](#_Toc165022122)

[Replication with Shortened Group Lists 28](#_Toc165022123)

[Replication without health-related groups 31](#_Toc165022124)

[Controlling for polysemy, frequency, and semantic drift 32](#_Toc165022125)

[Full table of estimated change across groups 35](#_Toc165022126)

# Additional details on text data sources

Hamilton and colleagues (2016) trained word embeddings on large-scale English-language books obtained from Google Books and the Corpus of Historical American English (COHA). Embeddings were trained using *word2vec* with skip-gram with negative sampling (*SGNS*); further details on *word2vec* and *SGNS* embeddings are provided at length elsewhere (for an introduction, see supplemental materials in Charlesworth et al., 2021, 2022). Hamilton and colleagues (2016) also specified standard hyperparameters for *word2vec* training: 300-dimensional embeddings (i.e., every embedding is a vector of 300 numbers), with symmetric context windows of 4 words on either side of the target word. Embeddings were computed only for words that passed a frequency cut-off of at least 500 observations in a given decade (due to its smaller size, the frequency cut-off for COHA was 100 observations in a given decade). Further specifications on model training and the underlying corpora can be found in the original manuscript of Hamilton and colleagues (2016).

Although Hamilton and colleagues (2016) provide data from six datasets of historical text including both English and non-English corpora (from German, French, and simplified Chinese), the aims of the current project are focused on the largest and most representative English-language corpora of (1) *EngAll*, the primary corpus of English-language fiction and non-fiction books extracted from Google n-grams (specifically, 5-grams, or 5-word fragments), which spans text from 1800-1999 and includes approximately 850 billion word tokens over this time span; and (2) *COHA*, a genre-balanced corpus from 1810 – 2009 and approximately 410 million word tokens. Many of the stigmatized groups we study are only present starting around the 1900s; thus, we restricted our focus to the EngAll and COHA data from 1900-2000.

# Stigma selection process

As noted in the main text, our process of selecting the sample space of stigmatized identities began with a list of 93 stigmatized identities, characteristics, and statuses (Pachankis et al., 2017). This list was then further subset to remove any stigmatized groups that had overlapping words (e.g., cancer remitted, breast cancer, lung cancer, and so on), and then further removed any groups that could not be adequately represented in synonyms that had at least one word available across the 10 decades investigated. Ultimately, these relatively expansive inclusion criteria led us to a final sample of 58 stigmatized groups (Table S1).

For each of the final 58 stigmatized groups, we generated lists of single word synonyms using both historical and contemporary thesauruses (e.g., *Oxford Historical Thesaurus, Thesaurus.com*). Table S1 lists the chosen synonyms for each of the 58 groups. From these lists, we also identified short lists of labels that captured only the most central and unambiguous group-related concept. The short lists were used in supplementary robustness analyses reported later in this document; some groups could be argued to show such clear short lists (see Table S1), resulting in a sample of 43 groups for that analysis. Additionally, for the COHA analyses, not all groups had at least one label available across decades, resulting in a sample of 42 groups for COHA analyses because 16 groups did not have sufficient synonyms across time.

**Table S1**.

Group label synonyms available for 58 stigmatized group representations

| **Group** | **Full list of labels** | **Shortened list of central labels** |
| --- | --- | --- |
| **aboriginal** | aboriginal aboriginals native aborigine inuit indigenous natives eskimo navajo pueblo apache sioux cherokee hopi comache algonquin shawnee pawnee lakota pima alaskan cheyenne | aboriginals natives indigenous aborigine |
| **abortion** | abortion misbirth aborted abort abortionist prorights unborn | -- |
| **alcoholic** | alcoholic drinker alcoholism intoxicated alcohol drinking intoxication drunk drunkard | alcoholics drunkards drinkers drunks |
| **middleeastern** | arabian arabians arab arabs turk turks turkish persia persian persians kurd kurds kurdish azerbaijani azerbaijanis iranian iranians | arabs turks persians iranians |
| **asexual** | asexual asexuals nonsexual celibacy celibate chastity chaste sexless asexuality asexy sexfree agamous | asexuals nonsexual celibate celibates |
| **asian** | asian asians japanese chinese korean taiwanese tibetan phillipino mongolian tibetan bangladeshi bhutanese indian nepalese pakistani burmese cambodian filipino indonesian malaysian thai | asians chinese korean japanese |
| **atheist** | atheist atheists infidel infidels secular agnostic godless atheism ungodly heathen heathens | atheists infidels atheist infidel |
| **heartattack** | heartattack tachycardia palpitation cardiac hypertension bypass coronary pulmonary vascular cardiovascular | -- |
| **bipolar** | bipolar manic manics mania depressed depressive depression psychotic psychosis | bipolar mania psychosis manic |
| **black** | black african africans blacks colored coloreds negro negros nigger niggers nigga niggas afro afros | blacks negros africans african |
| **blind** | blind visionless blindness blinded impaired unseeing sightless | blind visionless blindness unseeing |
| **cancer** | cancer chemo chemotherapy carcinoma carcinogen tumor biopsy malignant cancerous tumour malignancy cancerpatient | -- |
| **christian** | christian christians fundamentalist fundamentalists catholic catholics evangelical evangelicals baptist baptists christianity protestant protestants lutheran lutherans methodist methodists | christians catholics protestants evangelicals |
| **criminal** | criminal criminals inmate inmates offender offenders felon felons incarcerated perpetrator perpetrators convict convicts convicted imprisoned suspect suspects arrested jailed guilty indicted | criminals felons convicts offenders |
| **deaf** | deaf deafened deafness unhearing impaired earless hearingless | deaf deafness deafened unhearing |
| **dealer** | drugdealer dealer pusher peddler narcotic meth heroin cocaine trafficker | drugdealer dealer trafficker peddler |
| **depressed** | depressed sad depression suicidal sadness gloomy hopeless unhappy | depressed suicidal hopeless sadness |
| **diabetic** | diabetic hyperglycemic diabetes hyperglycemia hyperglycaemia polyphagia polydipsia mellitus diuresis | diabetic diabetes hyperglycemia mellitus |
| **disabled** | disabled cripple cripples crip crips disable handicapped disability | disabled cripples disability cripple |
| **divorced** | divorced divorces unmarried unhitched separated alimony estranged single | divorced divorcee unmarried divorce |
| **drugaddict** | drugaddict drugaddicts druggie druggies addict addicts addicted crackhead junkie junkies dopehead cocaine overdose overdosed coke dope narcotic meth heroin cannabis weed marijuana | addicts addicted junkie addict |
| **fat** | fat fatty weight chubby obese plump overweight tubby stout chunky heavy hefty potbelly potbellied pudgy | fat overweight chubby obese |
| **gang** | gangster mobster gangsters mobsters gang gangs thug thugs mafia mob mafioso mobs | gangster gangsters mobster mobsters |
| **herpes** | herpes herpetic gonorrhea syphilis venereal chlamydia warts | -- |
| **hiv** | hiv aids hivaids immunodeficient immunodeficiency | -- |
| **homeless** | homeless vagrant vagrants homelessness orphan orphans vagabond vagabonds | homeless vagrants vagabonds orphans |
| **gay** | homosexual homosexuals gay gays lesbian lesbians bisexual bisexuals queer queers lgbt transgender transgendered homo faggot fag transvestite tribads tribades sodomy sodomite sodomites homophile homophiles | homosexuals somodites gays lesbians |
| **immigrant** | immigrant immigrants migrant migrants newcomer newcomers emmigrant emmigrants noncitizen noncitizens alien aliens | immigrants emmigrants noncitizens aliens |
| **incontinent** | incontinent incontinence bedwetter bedwetters | -- |
| **indian** | indian indians pakistani pakistanis bangladeshi bangladeshis bengali gendalis hindu hindus gujarati punjabi nepalese nepali kashmiri tibetan gujaratis punjabis nepalis kashmiris tibetans | indians pakistanis hindus punjabis |
| **infertile** | infertile childless sterile infertility barren impotent unfertile infecund unbearing | childless infertile unfertile infertility |
| **jewish** | jewish jew jews judaism gentile gentiles zionist zionists synagogue torah bethelem hebrew semitic yiddish orthodox | jews jewish jew judaism |
| **laborer** | laborer laborers labourer labourers bluecollar craftsman craftsmen mechanic mechanics peasant farmer builder bricklayer | laborers laborer peasant farmer |
| **latino** | hispanic hispanics latino latinos latina latinas cuban cubans mexican mexicans spanish guatemalan honduran nicaraguan panamanian argentinian colombian brazilian venezuelan caribbean | hispanics latinos mexicans hispanic |
| **molested** | raped molested assaulted violated abused harassed | raped molested abused assaulted |
| **molestor** | molestor molestors rapist rapists harasser harassers offender offenders predator predators | molestor rapist rapists predators |
| **multiracial** | multiracial multiracials interracial multicultural multiculturals multiethnic biracial mulatto mulattos | multiracial multicultural interracial mulattos |
| **muslim** | muslim islamic muslims moslem moslems sunni sunnis shia shias islamist | muslims islamic muslim islam |
| **mute** | mute dumb muted aphasia aphonia mutism broca wernicke paraphasia silent muffled | mute muted dumb silent |
| **old** | old elderly elder elders older aged seniors grandparent grandparents grandmother grandmothers grandfather grandfathers | elderly elders grandparents senior |
| **paroled** | paroled pardoned pardon parole probation acquitted parolee | paroled pardoned probation parolee |
| **pierced** | pierced tattooed earring tattoo inking piercing tattooing piercings inked marked | pierced tattooed inked marked |
| **polygamous** | polygamous polygamy polyamory polyandry polygyny bigamy poly | polygamous polygamy polyandry poly |
| **poor** | poor beggar beggars needy wretch wretches impoverished destitute penniless unaffluent underprivileged | poor beggars needy penniless |
| **prostitute** | prostitute whore prostitutes whores prostitution courtesan escort hooker streetwalker streetworker | prostitutes whores prostitute whore |
| **psoriasis** | psoriasis inflammatory inflammation eczema dermatitis rosacea acne hives | -- |
| **retarded** | retarded stupid idiot idiots retard retards mental institutionalized | retarded idiot idiots retards |
| **scarred** | scarred wounded deformed bruised mutilated marred | scarred wounded deformed bruised |
| **schizophrenic** | schizophrenic schizophrenics schizophrenia demented dementia praecox psychotic psychotics psychosis | schizophrenics praecox psychosis schizophrenic |
| **server** | server servers waiter waitress waiters bartender bartenders cleaner cleaners maid maids | servers waiters bartenders maids |
| **short** | midget midgets dwarf dwarfs dwarfism | midgets dwarfs dwarf midget |
| **smoker** | smoker pothead smokers potheads cigarettes tobacco cigarette smoking | smokers smoker smoking cigarette |
| **std** | std sti syphilis aids hiv herpes chlamydia gonorrhoea gonorrhea | -- |
| **stroke** | stroke paralyzed hemorrhage dementia epilepsy eplieptic | -- |
| **transgender** | transgender transgenders transvestite transvestites tranny trannie shemale ladyboy trans genderqueer crossdressing crossdresser transsexual dysphoria twospirit hijra | transgender transvestite transvestites transsexual |
| **unattractive** | unattractive ugly awkward hideous grotesque unappealing unbeautiful unpretty unsightly | unattractive ugly grotesque hideous |
| **uneducated** | uneducated ignorant layman inexperienced illiterate illiterates unskilled untutored unknowledgeable untaught uninformed unread unlettered unschooled inerudite | uneducated ignorant uninformed illiterate |
| **unemployed** | unemployed jobless unemployment inactive idle | unemployed jobless unemployment idle |
| **wheelchair** | handicapped wheelchair paralysis paralyzed cripple handicap crippled disabled | wheelchair paralyzed paralysis handicapped |

*Note.* Groups with central synonym lists noted as “—” indicate those groups that do not have clear and unambiguous group-specific synonyms (e.g., std, hiv). As such, we excluded these groups in the robustness analysis with central labels only.

Table S2 indicates the mean, standard deviation, and minimum/maximum number of available group labels collapsing across all 58 stigmas for each decade. Although fewer group label synonyms were available in earlier decades, on average, we had 7.04 group labels to represent the stigmatized groups in EngAll (our main corpus of interest) and 3.12 group labels to represent the stigmatized groups in COHA (our replication corpus). Across EngAll and COHA, the notable difference in corpus size and number of available group labels makes the replication all the more noteworthy and shows the robustness of our general conclusion on stigma stability.

**Table S2**.

N available group label synonyms across all 58 groups, separating by corpus and decade

| **Decade** | **Books (Eng-All)** | | | |  | **COHA** | | | |
| --- | --- | --- | --- | --- | --- | --- | --- | --- | --- |
|  | **Mean *N*** | **SD *N*** | **Min *N*** | **Max *N*** |  | **Mean *N*** | **SD *N*** | **Min *N*** | **Max *N*** |
| 1900 | 5.90 | 3.52 | 1 | 19 |  | 2.41 | 2.38 | 0 | 9 |
| 1910 | 6.28 | 3.58 | 1 | 20 |  | 2.60 | 2.51 | 0 | 10 |
| 1920 | 6.34 | 3.45 | 1 | 19 |  | 3.00 | 2.71 | 0 | 12 |
| 1930 | 6.19 | 3.63 | 1 | 19 |  | 2.79 | 2.48 | 0 | 11 |
| 1940 | 5.98 | 3.48 | 1 | 18 |  | 2.74 | 2.49 | 0 | 9 |
| 1950 | 6.31 | 3.59 | 1 | 19 |  | 2.95 | 2.70 | 0 | 11 |
| 1960 | 7.00 | 3.83 | 1 | 20 |  | 3.02 | 2.67 | 0 | 10 |
| 1970 | 7.86 | 4.11 | 1 | 21 |  | 3.43 | 2.91 | 0 | 14 |
| 1980 | 8.33 | 4.00 | 2 | 21 |  | 3.66 | 2.92 | 0 | 12 |
| 1990 | 8.55 | 4.04 | 2 | 21 |  | 3.84 | 2.87 | 0 | 11 |
| 2000 | 8.71 | 4.16 | 2 | 21 |  | 3.91 | 2.99 | 0 | 12 |

# Computing stereotype content and latent valence, warmth, and competence

To calculate the stereotype content and latent meaning (valence, warmth, and competence) of stigmatized group representations, as well as their respective patterns of change, our procedure follows three primary steps. First, in ***Step One***, we extracted the top-10 absolute trait associates to a stigmatized group in a given decade. Specifically, using a list of 414 traits (all the traits that were available in EngAll from a larger list of over 600 traits), we first computed the average cosine similarity between a given target trait (e.g., “untrustworthy”) and all group label synonyms that were used to represent a group (e.g., *Dealer,* which was represented by synonyms including *dealer, peddler, narcotic, heroin, cocaine, supplier*). We then take the average of these cosine similarities [untrustworthy*-dealer,* untrustworthy*-peddler,* untrustworthy*-narcotic,* untrustworthy*-heroin,* untrustworthy*-cocaine,* and untrustworthy*-supplier*] to yield an average cosine similarity of untrustworthy-*Dealer*. We repeat this approach for all of the 414 traits available in the data. Next, we rank all traits according to their average cosine similarity to the given stigmatized group. For example, for *Dealer,* the top-10 ranked traits in 2000 were [compulsive, gullible, unethical, disorderly, nonchalant, unscrupulous, inept, antisocial, fickle, negligent].

Note that this approach for obtaining the top-10 trait associates deviates from previous papers (Charlesworth et al., 2022b, 2023) in that the current method does not require a comparison or contrast group. Rather, here we are looking at the top *absolute* associates to a stigmatized group independent of any other comparison group. This approach enables more flexibility when examining stigmatized groups that may not have direct comparison groups (e.g., the opposite of *Dealer* is not self-evident). Moreover, focusing on absolute associates also ensures that we are not artificially creating a scenario in which the negativity of group stereotypes will “cancel each other out” because we include both stigmatized groups and non-stigmatized comparison groups (e.g., if both *Black* and *White* were included, a stable population mean could arise because an increase in positive representations of Black is yoked to a decrease in positive representations of White). In short, this novel absolute approach is both more flexible and necessary for investigating the particular research question at hand.

Next, in ***Step Two,*** we replaced the top-10 absolute traits with their corresponding valence rating that was contextualized to that specific decade. Specifically, rather than assume that a single rating of valence (e.g., from valence rating norms) was applicable across 100 years (as we ourselves had done in previous work; Charlesworth et al., 2022b), we allowed the valence of traits to vary across time. To do so, we first created lists of 25 words that strongly (and stably) signaled positivity/negativity, based off the lists used for the *Implicit Association Test* and the *Word Embeddings Association Test*. Then, we took each trait (from the 414 traits) and examined the relative cosine similarity to 25 positive words compared to the cosine similarity to 25 negative words within each decade of text. Thus, each trait had a positivity/negativity score (its relative association to positive over negative seed words) in each decade. For example, in 1900, the trait *disagreeable* had the second-strongest negative association (val_histcontx_ = -0.26); in 2000, by contrast, the negativity of that trait had lessened to val_histcontx_ = -0.10, placing it as the 114^th^ most negative trait.

We used these historically-contextualized valence scores of each trait within a decade of text and took the average valence score across the top-10 traits within a decade. For example, imagine the top ranked traits for *Aboriginal* include [hostile, rebellious, adventurous, superstitious]. The corresponding historically-contextualized valence ratings for each of these traits in 1900 are [-0.13, -0.18, 0.05, -0.19] and in 2000 are [-0.14, -0.11, -0.02, -0.15]. Taking the average across these traits returns an average valence for *Aboriginal* of -0.11 in 1900 and -0.11 in 2000. We repeat this computation for all 11 decades (1900-2000) resulting in an 11-decade long timeseries of average historically-contextualized valence scores for each stigmatized group.

In an analogous way, we also compute the latent warmth and competence of the stereotypic representations, two of the most fundamental dimensions of stereotyping and person perception (Fiske et al., 2002). Again, we use a set of “anchor” words, drawn from the dictionaries provided by Nicolas and colleagues (2021), that represent the extreme poles of warmth/coldness and competence/incompetence. Then, each of the 414 available traits is assessed in relation to these lists of words and receives a score of its relative warmth/coldness or competence/incompetence within each decade of text. Finally, for each decade, we calculate the average warmth and competence score of the top-10 absolute traits. In the end, each group has a timeseries of 11 decades (1900-2000, inclusive) for each of these scores of latent valence, warmth, and competence. To calculate change, we estimate a mixed-effects Bayesian linear regression, allowing for random intercepts and random slopes by group. Results of the random slopes are presented in Table 1 of the main text.

As a final analytic step, in ***Step Three****,* we also consider the degree of turn-over in the top-10 traits themselves. Most simply, this could be calculated as the proportion of identical traits across time. That is, we are essentially calculating how many of the traits in 1900 repeat themselves in 1910. However, this neglects the fact that, among 414 traits, it is very likely that new traits may be used across time that are still of a very similar meaning (e.g., mean, unkind, unfriendly). Thus, building from advances that we introduced in recent work (Charlesworth et al., 2023), we instead compute the degree of turn-over in manifest trait content using cosine similarities *among* the traits across time. We do so by taking all pairwise cosine similarities between the top-10 traits in all pairs of successive decades, for example, 1900 and 1910 or 1910 and 1920 or 1920 and 1930, and so on. We then take the average successive-decade cosine similarity among traits, such that higher scores will indicate more similarity in the traits’ meaning across the two successive decades. Finally, we take the average of these successive-decade cosine similarities, and then take the inverse (1-*x*) of this average to get the degree of *dis*similarity across time. Ultimately, the result is a score of how much the manifest trait meaning of a group’s stereotype shifts across time, on average. Just as the slope estimates for valence, warmth, or competence give an understanding of how much change in *y* (e.g., valence) occurs from a one-unit change in *x* (time)*,* the manifest trait change score gives an index of how much change in *y* (trait content) occurs from a shift of one decade.

# Main models: Bayesian linear mixed effects models

Each of the 58 groups has an 11-decade long timeseries of average valence scores, resulting in a final sample of 638 observations, nested within 58 groups and 11 time points. To this dataset we fit a Bayesian mixed effects model with a uniform prior, random intercepts and random slopes for each group, and all other default parameters and model specifications (e.g., 2000 iterations, 4 chains) using *brms* in the R computing environment (*brms* package version 2.17.0). We chose to rely on the Bayesian model as our primary analysis because the posterior estimates of the population slope can provide a more direct quantification of the amount of evidence in favor of the null (i.e., a slope that is not meaningfully different from zero). For interpretation, we focus on the region of practical equivalence or ROPE (Kruschke, 2018), which is a pre-specified range of values that would reasonably be seen as indicating a “null effect.” As is standard, this range is typically set to +/- 0.1*SD_y_, although one can set more conservative thresholds (e.g., 0.05*SD_y_). For inference, we compute the percentage of the model’s full posterior that falls within the ROPE (for robustness we also check this at varying thresholds of ROPE and confirm that our interpretation is consistent across these thresholds).

Results from the model (reported in Table S3) show that it provides good fit to the data, with an R^2^ of 0.79 [0.77, 0.81]. Parameter estimates reveal, on average, a slight decrease in population-level or aggregate negativity across the 58 groups (Table S3). However, the highest density interval (HDI) is close to zero and, moreover, 100 percent of the model posterior falls within the standard ROPE, as well as a more conservative ROPE (0.05*SD_y_). Thus, we interpret the results as showing that stigmatized groups have been, for the most part, stable in negativity over 100 years of English-language book text.

**Table S3**.

Bayesian mixed effects model predicting valence from time: Population (aggregate) estimates.

|  | ***Estimate*** | ***Estimate Error*** | ***Lower 95% HDI*** | ***Upper 95% HDI*** | ***% in ROPE***  ***(0.1*SD_y_)*** |
| --- | --- | --- | --- | --- | --- |
| ***Intercept*** | -0.059 | 0.0091 | -0.077 | -0.041 | 0% |
| **Time** | -0.0030 | 0.00063 | -0.0042 | -0.0017 | 100% |

As a mixed effect model, the model also specifies individual group-level random slopes. From these data, we can build our understanding of the complementary mechanisms that might be upholding such net stigma negativity. First, to understand the prevalence of reproducibility versus replacement, we look at how many groups are, themselves, stable in trajectories across time, which is empirically in line with the reproducibility mechanism. Table S4 (reproducing some data also reported as Table 1 in the main text) provides individual group intercepts and slopes for all 58 groups, as well as a classification of whether the group’s slope HDI includes zero.

We can see, first, meaningful variation in the slopes across groups, ranging from the strongest negative slopes, indicating more negative representations across time of -0.13 (for *HIV*) and -0.12 (for *Asexual*), to positive slopes that are weaker in magnitude of 0.0026 (for *Atheist*) and 0.0025 (for *Criminal*), which generally reflect groups that were initially very negative and became slightly more positively represented over time. In other words, we see variation, with some groups increasing and other groups decreasing in negativity. At the same time, over half of these group random slope estimates are small; as reported in the main text, 33 out of 58 groups (57%) have HDIs for the random slopes that include zero. Thus, while there is some variance across groups, there is also a general pattern of stability, such that most individual groups are not substantively changing in negativity even across 100 years of text. Notably, for the remaining 43% of groups that *are* showing some degree of change, all of them are changing towards more negative representations over time; this result is in line with the finding of the slight negative slope observed in the aggregate analyses as well.

**Table S4**.

Primary Bayesian mixed effects model predicting latent valence from time: Random slopes and intercepts across 58 groups

|  | ***Group intercept*** | ***Group intercept lower 95% HDI*** | ***Group intercept upper 95% HDI*** | ***Group slope*** | ***Group slope lower 95% HDI*** | ***Group intercept upper 95% HDI*** | ***Slope HDI includes 0?*** |
| --- | --- | --- | --- | --- | --- | --- | --- |
| aboriginal | -0.0469 | -0.0736 | -0.0202 | -0.0046 | -0.0081 | -0.0011 | Not zero |
| abortion | -0.0845 | -0.1114 | -0.0578 | -0.0023 | -0.0060 | 0.0012 | Includes zero |
| alcoholic | -0.0943 | -0.1210 | -0.0676 | -0.0023 | -0.0059 | 0.0012 | Includes zero |
| asexual | 0.0509 | 0.0238 | 0.0789 | -0.0121 | -0.0160 | -0.0085 | Not zero |
| asian | 0.0054 | -0.0227 | 0.0326 | -0.0043 | -0.0079 | -0.0006 | Not zero |
| atheist | -0.1482 | -0.1745 | -0.1213 | 0.0026 | -0.0009 | 0.0062 | Includes zero |
| bipolar | -0.0843 | -0.1104 | -0.0570 | -0.0019 | -0.0055 | 0.0015 | Includes zero |
| black | -0.0760 | -0.1029 | -0.0491 | 0.0005 | -0.0030 | 0.0042 | Includes zero |
| blind | -0.1277 | -0.1530 | -0.1020 | 0.0005 | -0.0028 | 0.0038 | Includes zero |
| cancer | -0.0594 | -0.0862 | -0.0341 | -0.0018 | -0.0051 | 0.0016 | Includes zero |
| christian | 0.0300 | 0.0030 | 0.0581 | -0.0055 | -0.0091 | -0.0018 | Not zero |
| criminal | -0.1699 | -0.1963 | -0.1422 | 0.0025 | -0.0011 | 0.0061 | Includes zero |
| deaf | -0.1153 | -0.1428 | -0.0887 | 0.0006 | -0.0030 | 0.0042 | Includes zero |
| dealer | -0.0223 | -0.0484 | 0.0042 | -0.0071 | -0.0108 | -0.0036 | Not zero |
| depressed | -0.1284 | -0.1557 | -0.1027 | 0.0009 | -0.0027 | 0.0045 | Includes zero |
| diabetic | -0.0414 | -0.0686 | -0.0148 | -0.0044 | -0.0079 | -0.0009 | Not zero |
| disabled | -0.1296 | -0.1569 | -0.1020 | 0.0023 | -0.0011 | 0.0058 | Includes zero |
| divorced | -0.0172 | -0.0442 | 0.0098 | -0.0028 | -0.0064 | 0.0008 | Includes zero |
| drugaddict | -0.0674 | -0.0940 | -0.0419 | -0.0042 | -0.0077 | -0.0007 | Not zero |
| fat | -0.0063 | -0.0340 | 0.0207 | -0.0056 | -0.0091 | -0.0020 | Not zero |
| gang | -0.1256 | -0.1521 | -0.0993 | 0.0010 | -0.0025 | 0.0045 | Includes zero |
| gay | 0.0178 | -0.0093 | 0.0446 | -0.0095 | -0.0132 | -0.0060 | Not zero |
| heartattack | -0.0554 | -0.0823 | -0.0292 | -0.0023 | -0.0058 | 0.0012 | Includes zero |
| herpes | -0.0743 | -0.1005 | -0.0486 | -0.0027 | -0.0061 | 0.0008 | Includes zero |
| hiv | 0.0867 | 0.0594 | 0.1145 | -0.0132 | -0.0170 | -0.0095 | Not zero |
| homeless | -0.1187 | -0.1459 | -0.0918 | 0.0002 | -0.0032 | 0.0038 | Includes zero |
| immigrant | -0.0249 | -0.0503 | 0.0019 | -0.0058 | -0.0093 | -0.0024 | Not zero |
| indian | -0.0395 | -0.0669 | -0.0132 | -0.0043 | -0.0078 | -0.0008 | Not zero |
| infertile | -0.1317 | -0.1580 | -0.1054 | 0.0014 | -0.0020 | 0.0049 | Includes zero |
| jewish | 0.0042 | -0.0231 | 0.0307 | -0.0044 | -0.0079 | -0.0008 | Not zero |
| laborer | 0.1079 | 0.0809 | 0.1347 | -0.0117 | -0.0153 | -0.0081 | Not zero |
| latino | -0.0161 | -0.0433 | 0.0112 | -0.0052 | -0.0087 | -0.0017 | Not zero |
| middleeastern | -0.1119 | -0.1401 | -0.0852 | 0.0024 | -0.0011 | 0.0062 | Includes zero |
| molested | -0.1512 | -0.1780 | -0.1244 | 0.0018 | -0.0016 | 0.0053 | Includes zero |
| molestor | -0.1275 | -0.1542 | -0.1008 | 0.0001 | -0.0035 | 0.0036 | Includes zero |
| multiracial | -0.0134 | -0.0417 | 0.0152 | -0.0053 | -0.0089 | -0.0016 | Not zero |
| muslim | -0.0544 | -0.0824 | -0.0280 | -0.0005 | -0.0040 | 0.0033 | Includes zero |
| mute | -0.0754 | -0.1018 | -0.0501 | -0.0015 | -0.0049 | 0.0019 | Includes zero |
| old | 0.0951 | 0.0672 | 0.1218 | -0.0080 | -0.0116 | -0.0041 | Not zero |
| paroled | -0.0439 | -0.0700 | -0.0178 | -0.0044 | -0.0079 | -0.0011 | Not zero |
| pierced | -0.0308 | -0.0563 | -0.0040 | -0.0060 | -0.0094 | -0.0026 | Not zero |
| polygamous | -0.1118 | -0.1372 | -0.0857 | 0.0002 | -0.0031 | 0.0035 | Includes zero |
| poor | -0.1213 | -0.1487 | -0.0951 | 0.0012 | -0.0024 | 0.0049 | Includes zero |
| prostitute | -0.0932 | -0.1194 | -0.0666 | -0.0014 | -0.0048 | 0.0020 | Includes zero |
| psoriasis | -0.0628 | -0.0892 | -0.0362 | -0.0039 | -0.0075 | -0.0005 | Not zero |
| retarded | -0.0863 | -0.1125 | -0.0606 | -0.0029 | -0.0065 | 0.0005 | Includes zero |
| scarred | -0.1400 | -0.1657 | -0.1142 | 0.0002 | -0.0033 | 0.0036 | Includes zero |
| schizophrenic | -0.0625 | -0.0890 | -0.0366 | -0.0030 | -0.0064 | 0.0004 | Includes zero |
| server | 0.0436 | 0.0161 | 0.0712 | -0.0091 | -0.0128 | -0.0055 | Not zero |
| short | -0.0835 | -0.1094 | -0.0573 | -0.0015 | -0.0050 | 0.0020 | Includes zero |
| smoker | -0.0140 | -0.0402 | 0.0123 | -0.0060 | -0.0096 | -0.0026 | Not zero |
| std | -0.0397 | -0.0664 | -0.0138 | -0.0042 | -0.0076 | -0.0008 | Not zero |
| stroke | -0.0746 | -0.0997 | -0.0495 | -0.0020 | -0.0054 | 0.0014 | Includes zero |
| transgender | 0.0455 | 0.0184 | 0.0735 | -0.0091 | -0.0128 | -0.0056 | Not zero |
| unattractive | -0.1383 | -0.1653 | -0.1124 | 0.0005 | -0.0031 | 0.0041 | Includes zero |
| uneducated | -0.0443 | -0.0698 | -0.0178 | -0.0048 | -0.0084 | -0.0014 | Not zero |
| unemployed | -0.0929 | -0.1196 | -0.0672 | -0.0009 | -0.0043 | 0.0025 | Includes zero |
| wheelchair | -0.1031 | -0.1297 | -0.0764 | -0.0007 | -0.0043 | 0.0028 | Includes zero |

# Additional details on mechanism of replacement: Correlations across groups

In the main text (Figure 2), we propose a complementary mechanism of “replacement” in which changing negativity towards a target group may nevertheless transfer to other, secondary group(s). One empirical form of replacement – “semantic transfer” – would occur when groups are replacing one another in stigma through some shared semantic space, such that groups with similar trait spaces should experience more “transfer” than groups that are very different in their trait spaces. This means that, given a group changing in stigma negativity (e.g., *Asexual,* which has become more negative), we would expect a “transfer” to occur towards groups with high correlations in semantic spaces at time t_1_ (1900) and predict the slopes from t_1_ to t_n_. Note that this can transfer could theoretically occur even in the current setting where there are no groups with meaningful positive slopes; rather, what is required is simply that the semantic relationships between groups predict greater differences in the patterns of change between groups (e.g., as one strengthens in negativity, a second group lessens in negativity).

A second form of replacement – “non-semantic transfer” – is based on the idea that any group could suffice as a new target for negativity, regardless of its semantic relationship to the changing group. In this case, given a group changing in stigma negativity, we would expect no significant relationship between cross-group semantic correlations and similarity in the degree of change. Instead, the reason for the similarity in change is likely attributable to some other factor, such as shared physical location or similar prevalence in the environment. Empirically, this second pattern would result in a null correlation between semantic similarity and the difference in change slopes across groups.

As briefly discussed in the main text, we test these two replacement patterns as follows. First, taking the full trait spaces of 414 available traits, we examine cross-group correlations in trait-to-group cosine similarities. For example, looking at the vector of cosines of all traits to the group *Alcoholic,* we compute the correlation of that vector with the vector of cosines for all traits to the group *Drug Addict* or *Homeless*. We perform this cross-group correlation analysis within each decade. The result is therefore a 58 group x 58 group correlation matrix for each decade, with higher scores indicating more similar trait-group representations.

Figures S1 and S2 below show the correlation matrices for decades 1900 and 2000, respectively. Similar results of group clusters are seen across both decades, even 100 years apart. For instance, across decades, the top left corner of the correlograms shows that there is a health/sexuality stigma cluster including stigmas of [*Transgender, HIV, STD*], as well as clusters with groups perceived to have high threat including [*Black, Criminal, Schizophrenic*], a disability-specific cluster, a poverty cluster, and so on. In short, the clustering based on bottom-up semantic content returns the expected structure of cross-group relations which can, in turn, be used to predict how stigma for one group may transfer over to a second group.

***Figure S1.* Correlations among group representations in 1900.** Groups are ordered according to default hierarchical clustering in the *corrplot* package in R. Darker blue indicates stronger positive correlations across groups (i.e., correlations among their vectors of group-trait associations) and thus more similarity in representations; darker red indicates stronger negative correlations across groups and thus more dissimilarity in representations. Clusters of groups are indicated by “boxes” of blue positive correlations, meaning that all groups in the box share trait spaces.

***Figure S2.* Correlations among group representations in 2000.** Groups are ordered according to the order in 1900, which was calculated using default hierarchical clustering in the *corrplot* package in R. Darker blue indicates stronger positive correlations across groups (i.e., correlations among their vectors of group-trait associations) and thus more similarity in representations; darker red indicates stronger negative correlations across groups and thus more dissimilarity in representations.

From these cross-group correlations, we next examine the relationship between cross-group semantic relationships and transfer of change. To do so, we first identify a target group (e.g., *Asexual*) and then pull the t_1_ cross-group correlations of that target group with the 57 other groups, resulting in 57 estimates of how much the target group is related to the remaining groups. Second, we create a difference estimate of how similar each pair of groups are in their patterns of change, by taking the absolute value of the difference between the transfer group’s slope minus the target group’s slope. Larger numbers here would indicate that the transfer group’s slope is going in the exact opposite direction as the target group’s slope; in contrast, smaller numbers would indicate that the transfer and target groups are very similar.

Across all the 25 changing groups (identified in the primary Bayesian regression reported in Study 1), we calculate how many show the patterns of (1) replacement via shared semantics (positive correlations); (2) replacement via processes other than shared semantics (null relationships); or (3) a generalization pattern (discussed in the main text). Empirically, this latter pattern of change would result in a negative correlation between the groups’ semantic similarity and the difference in slopes between groups, because the more semantically similar the groups, the *less* different their slopes (i.e., the more the change should generalize between similar groups).

We use a simple cut-off of *rs* = [-0.2, 0.2] (based on standard effect size heuristics), with values inside the range indicating null relationships, values above the range indicating a positive correlation, or below the range indicating a negative correlation. As reported in the main text, the majority of groups show null relationships, with a handful of five groups showing negative relationships that would suggest generalization (Figure S3). Only one group (*Asexual*) shows a positive correlation that would suggest replacement via shared semantics (Figure S4). In short, based on these simple cut-offs, to the extent that transfer (replacement) may exist, it is likely doing so via non-semantic relationships rather than through shared semantic spaces.

***Figure S3.*** **Pearson correlation magnitudes between semantic similarity (in 1900) and differences in slope estimates for each listed target group (the 25 changing target groups).** Red points indicate those target groups that had negative correlations between cross-group semantic similarity in 1900 and cross-group slope dissimilarity (in other words, they showed “generalization”, because more semantic similarity corresponded to less dissimilarity in change). Blue points indicate those target groups that had positive correlations between semantic similarity in 1900 and cross-group slope dissimilarity (in other words, they showed a hydraulic “semantic transfer”, such that more semantic similarity corresponded to more dissimilarity in change). Grey points indicate all groups with null correlations, interpreted as groups that may have transferred negativity via non-semantic relationships.

| **A** | **B** | **C** |
| --- | --- | --- |
|  | | |

**Figure S4. Relationships between cross-group semantic similarity and similarity in slopes for Asexual (A), Fat (B), and Immigrant (C)*.*** These three group targets were chosen as case studies to demonstrate possible empirical patterns of replacement (or generalization of change). Y-axis represents the difference in the slope estimates between the target group and all 57 other potential secondary “transfer” groups. X-axis represents the semantic similarity between the target group and all 57 other potential secondary transfer groups in 1900.

# Replication of main analyses with frequentist models

While a Bayesian model with a uniform prior (as fitted for our primary model) will yield the same results as a frequentist model, we nevertheless offer a robustness test to demonstrate the consistency for those readers more familiar with frequentist models and null-hypothesis significance testing. Here, the frequentist model is fitted using *lmer* from the *lme4* package in *R* (package version 1.1-26). As with the Bayesian models, we find a substantial negative intercept in aggregate, population-level stereotypes of stigmatized groups, *b* = -0.059, *SE* = 0.0090, that has also slowly but significantly moved towards more negative representations, *b* = -0.0030, *SE* = 0.00063. Comparing results to those reported in Table S2 above, we can see the similarity of estimates and thus also the robustness of conclusions to these slight modeling choices.

# Replication with COHA dataset

A primary concern when assessing changes in stigma representations across time is that the underlying sample of corpora may shift across time as well. To that end, we make use of a *genre-balanced* corpus – the Corpus of Historical American English (COHA) – meaning that the same proportion of text genres (namely fiction and non-fiction) are present across all historical timesteps. Although it has a major advantage of being genre balanced, COHA is also much smaller (less than 1% the size of Google Books). Thus, we restricted our focus to 42 groups that were available with sufficient group label synonyms from 1900-2000. If we find that the general conclusions hold even for this smaller, genre-balanced corpus, then we will have greater confidence in the robustness of our results as reflecting generalizable patterns of historical stereotypes.

Indeed, results from the Bayesian mixed effects models show that the main conclusions were replicated in the COHA dataset (Figure S5). As above, the intercept was negative, *b =* -0.033, 95% HDI = [-0.043, -0.023], and the estimated posterior of the population-level slope for COHA data was small, *b* = -0.00075, 95% HDI = [-0.0018, 0.00027], with 100% of the posterior estimates falling within the standard ROPE. Despite this general consistency, COHA suggested slightly poorer fit from the model, *R^2^* = 0.32, 95% HDI = [0.24, 0.38], likely because the smaller size of COHA produces more volatility that decreases model performance.

***Figure S5.* Corpus of Historical American English (COHA) raw values of valence.** Thick black line indicates the average, net trajectory across all groups; thin colored lines indicate the individual raw trajectories for 42 stigmatized groups, as listed in Table S5.

Additionally, our second general conclusion regarding the dominant prevalence of a reproducibility mechanism was replicated with this alternative dataset. Again, nearly every single group’s slope (41/42 or 98%) had highest density intervals (HDIs) that included zero. The only changing group was *Gay,* which was likely changing in large part due to known semantic drift in the labels themselves, a process we address more directly in later supplemental analyses (see below). Thus, from the COHA analyses we conclude that (1) there is generally aggregate stability in the level of stereotype negativity towards stigmatized groups and (2) this aggregate stability comes in large part from stigma reproducibility mechanisms operating *within* groups (as suggested by the majority of groups showing zero slopes).

**Table S5**.

Corpus of Historical American English (COHA) data: Bayesian mixed effects model predicting valence from time: Random effects.

|  | ***Group intercept*** | ***Group intercept lower 95% HDI*** | ***Group intercept upper 95% HDI*** | ***Group slope*** | ***Group slope lower 95% HDI*** | ***Group intercept upper 95% HDI*** | ***Slope HDI includes 0?*** |
| --- | --- | --- | --- | --- | --- | --- | --- |
| **aboriginal** | -0.0210 | -0.0457 | 0.0062 | -0.0018 | -0.0052 | 0.0009 | Includes zero |
| **alcoholic** | -0.0528 | -0.0795 | -0.0278 | 0.0002 | -0.0026 | 0.0035 | Includes zero |
| **asian** | -0.0159 | -0.0401 | 0.0097 | -0.0016 | -0.0047 | 0.0013 | Includes zero |
| **atheist** | -0.0287 | -0.0554 | -0.0041 | -0.0003 | -0.0031 | 0.0031 | Includes zero |
| **bipolar** | -0.0542 | -0.0797 | -0.0275 | -0.0008 | -0.0042 | 0.0021 | Includes zero |
| **black** | -0.0267 | -0.0513 | -0.0010 | -0.0011 | -0.0041 | 0.0018 | Includes zero |
| **blind** | -0.0725 | -0.0997 | -0.0476 | 0.0010 | -0.0018 | 0.0046 | Includes zero |
| **christian** | -0.0058 | -0.0306 | 0.0208 | -0.0022 | -0.0056 | 0.0006 | Includes zero |
| **criminal** | -0.0431 | -0.0697 | -0.0183 | 0.0001 | -0.0027 | 0.0035 | Includes zero |
| **deaf** | -0.0561 | -0.0818 | -0.0302 | -0.0002 | -0.0032 | 0.0028 | Includes zero |
| **dealer** | -0.0188 | -0.0444 | 0.0074 | -0.0014 | -0.0046 | 0.0016 | Includes zero |
| **depressed** | -0.0645 | -0.0908 | -0.0392 | 0.0002 | -0.0029 | 0.0033 | Includes zero |
| **disabled** | -0.0327 | -0.0600 | -0.0078 | 0.0000 | -0.0027 | 0.0036 | Includes zero |
| **divorced** | -0.0054 | -0.0309 | 0.0213 | -0.0019 | -0.0051 | 0.0010 | Includes zero |
| **drugaddict** | -0.0414 | -0.0676 | -0.0169 | -0.0001 | -0.0028 | 0.0031 | Includes zero |
| **fat** | -0.0407 | -0.0666 | -0.0163 | -0.0001 | -0.0029 | 0.0030 | Includes zero |
| **gang** | -0.0411 | -0.0678 | -0.0167 | 0.0001 | -0.0027 | 0.0034 | Includes zero |
| **gay** | 0.0355 | 0.0006 | 0.0738 | -0.0055 | -0.0109 | -0.0009 | Not zero |
| **homeless** | -0.0240 | -0.0521 | 0.0013 | 0.0000 | -0.0028 | 0.0036 | Includes zero |
| **immigrant** | -0.0339 | -0.0587 | -0.0092 | -0.0009 | -0.0039 | 0.0019 | Includes zero |
| **indian** | -0.0174 | -0.0427 | 0.0076 | -0.0012 | -0.0042 | 0.0018 | Includes zero |
| **infertile** | -0.0502 | -0.0753 | -0.0243 | -0.0008 | -0.0041 | 0.0020 | Includes zero |
| **jewish** | -0.0218 | -0.0465 | 0.0034 | -0.0013 | -0.0043 | 0.0016 | Includes zero |
| **laborer** | -0.0058 | -0.0314 | 0.0209 | -0.0018 | -0.0050 | 0.0012 | Includes zero |
| **latino** | -0.0208 | -0.0462 | 0.0050 | -0.0011 | -0.0042 | 0.0019 | Includes zero |
| **middleeastern** | -0.0276 | -0.0527 | -0.0018 | -0.0014 | -0.0046 | 0.0014 | Includes zero |
| **molested** | -0.0493 | -0.0754 | -0.0249 | 0.0002 | -0.0025 | 0.0034 | Includes zero |
| **mute** | -0.0642 | -0.0921 | -0.0389 | 0.0008 | -0.0021 | 0.0043 | Includes zero |
| **old** | 0.0077 | -0.0198 | 0.0347 | -0.0012 | -0.0045 | 0.0025 | Includes zero |
| **paroled** | -0.0122 | -0.0372 | 0.0141 | -0.0016 | -0.0047 | 0.0013 | Includes zero |
| **pierced** | -0.0478 | -0.0728 | -0.0236 | -0.0001 | -0.0031 | 0.0029 | Includes zero |
| **poor** | -0.0445 | -0.0716 | -0.0193 | 0.0002 | -0.0026 | 0.0036 | Includes zero |
| **prostitute** | -0.0245 | -0.0487 | 0.0006 | -0.0011 | -0.0040 | 0.0017 | Includes zero |
| **retarded** | -0.0469 | -0.0722 | -0.0207 | -0.0010 | -0.0043 | 0.0018 | Includes zero |
| **scarred** | -0.0840 | -0.1121 | -0.0577 | 0.0014 | -0.0016 | 0.0051 | Includes zero |
| **server** | -0.0148 | -0.0410 | 0.0104 | -0.0006 | -0.0035 | 0.0028 | Includes zero |
| **smoker** | -0.0298 | -0.0551 | -0.0043 | -0.0009 | -0.0039 | 0.0021 | Includes zero |
| **stroke** | -0.0581 | -0.0834 | -0.0320 | -0.0005 | -0.0038 | 0.0025 | Includes zero |
| **unattractive** | -0.0415 | -0.0672 | -0.0161 | -0.0003 | -0.0032 | 0.0028 | Includes zero |
| **uneducated** | -0.0352 | -0.0606 | -0.0055 | -0.0022 | -0.0063 | 0.0007 | Includes zero |
| **unemployed** | -0.0234 | -0.0489 | 0.0016 | -0.0007 | -0.0036 | 0.0024 | Includes zero |
| **wheelchair** | -0.0500 | -0.0755 | -0.0255 | -0.0001 | -0.0029 | 0.0030 | Includes zero |

# Replication with Shortened Group Lists

Yet another concern when assessing group stereotypes in text is the fact that, ultimately, a researcher must decide on the best words to represent the group itself. In the primary analyses, we chose lists of words gathered from historical thesauruses to represent each group concept. The longer the list of group labels, the better we can comprehensively capture the group concept when we average across the associations with all group labels. At the same time, the longer the list of group labels, the more we sacrifice the *specificity* of the group concept and the more likely we are to pick up additional unintended or adjacent meanings beyond the group concept. Thus, to assess whether our conclusions were affected by the choice of using more comprehensive lists (vs. short, specific lists) of group labels, we replicated all analyses with each group represented by only 4 central synonyms. Given the higher criteria to have clear and unambiguous group-related labels, our sample size here was reduced to 43 groups (see Table 1 in the main text for the 43 groups and their 4 group labels).

Results show that the main conclusions, of aggregate stability and the dominance of reproducibility, were replicated even with this smaller sample of groups and with a different method of operationalizing group representations. As in the main analyses, the intercept of group representations was negative, *b* = -0.063, 95% HDI = [-0.084, -0.042], and the change (slope) was small but trending towards more negative representations across time, *b* = -0.0019, 95% HDI = [-0.0033, -0.00059], with 100% of the posterior estimates falling within the standard ROPE. Additionally, as with the main analyses of all 58 groups in the Google Books dataset, we found that the model provided good fit to the data, *R^2^* = 0.81, 95% HDI = [0.69, 0.83]. Turning to the individual random slopes for the 43 groups, we found that the majority (31/43 or 72%) were not meaningfully changing, again pointing to the prevalence of the reproducibility mechanism (Table S6). The remaining 12/43 (28%) groups indicated some change, and all changing towards more negative representations across time.

***Figure S6.* Google Books’ raw values of valence, with each group represented using only 4 central synonyms.** Thick black line indicates the average, net trajectory across all groups; thin colored lines indicate the individual raw trajectories for 43 stigmatized groups listed in Table S6.

**Table S6**.

Shortened group lists: Bayesian mixed effects model predicting latent valence from time

|  | ***Group intercept*** | ***Group intercept lower 95% HDI*** | ***Group intercept upper 95% HDI*** | ***Group slope*** | ***Group slope lower 95% HDI*** | ***Group intercept upper 95% HDI*** | ***Slope HDI includes 0?*** |
| --- | --- | --- | --- | --- | --- | --- | --- |
| aboriginal | -0.0282 | -0.0545 | -0.0019 | -0.0030 | -0.0066 | 0.0006 | Includes zero |
| alcoholic | -0.0955 | -0.1223 | -0.0691 | -0.0019 | -0.0055 | 0.0018 | Includes zero |
| asian | -0.0033 | -0.0305 | 0.0231 | -0.0022 | -0.0058 | 0.0015 | Includes zero |
| atheist | -0.1450 | -0.1724 | -0.1184 | 0.0025 | -0.0011 | 0.0063 | Includes zero |
| bipolar | -0.0699 | -0.0968 | -0.0431 | -0.0031 | -0.0068 | 0.0005 | Includes zero |
| black | -0.0861 | -0.1129 | -0.0596 | 0.0007 | -0.0029 | 0.0044 | Includes zero |
| blind | -0.1194 | -0.1463 | -0.0927 | 0.0002 | -0.0035 | 0.0037 | Includes zero |
| christian | -0.0632 | -0.0903 | -0.0363 | 0.0008 | -0.0027 | 0.0046 | Includes zero |
| criminal | -0.1559 | -0.1826 | -0.1292 | 0.0012 | -0.0025 | 0.0047 | Includes zero |
| deaf | -0.1139 | -0.1407 | -0.0866 | 0.0011 | -0.0025 | 0.0048 | Includes zero |
| dealer | 0.0418 | 0.0138 | 0.0700 | -0.0102 | -0.0143 | -0.0063 | Not zero |
| depressed | -0.1227 | -0.1491 | -0.0960 | -0.0005 | -0.0042 | 0.0031 | Includes zero |
| diabetic | -0.0434 | -0.0700 | -0.0163 | -0.0039 | -0.0076 | -0.0003 | Not zero |
| disabled | -0.1258 | -0.1519 | -0.0991 | 0.0013 | -0.0022 | 0.0048 | Includes zero |
| divorced | 0.0098 | -0.0168 | 0.0369 | -0.0057 | -0.0094 | -0.0021 | Not zero |
| drugaddict | -0.1043 | -0.1309 | -0.0786 | -0.0015 | -0.0051 | 0.0021 | Includes zero |
| fat | 0.0003 | -0.0270 | 0.0273 | -0.0078 | -0.0117 | -0.0040 | Not zero |
| homeless | -0.1305 | -0.1573 | -0.1040 | 0.0015 | -0.0021 | 0.0051 | Includes zero |
| immigrant | -0.0138 | -0.0403 | 0.0134 | -0.0056 | -0.0092 | -0.0019 | Not zero |
| indian | -0.0518 | -0.0786 | -0.0250 | -0.0015 | -0.0051 | 0.0022 | Includes zero |
| infertile | -0.0158 | -0.0421 | 0.0110 | -0.0057 | -0.0094 | -0.0022 | Not zero |
| jewish | -0.0079 | -0.0343 | 0.0190 | -0.0015 | -0.0052 | 0.0022 | Includes zero |
| laborer | 0.0819 | 0.0557 | 0.1082 | -0.0066 | -0.0102 | -0.0029 | Not zero |
| latino | -0.0448 | -0.0717 | -0.0181 | 0.0000 | -0.0037 | 0.0037 | Includes zero |
| middleeastern | -0.1105 | -0.1375 | -0.0842 | 0.0015 | -0.0020 | 0.0053 | Includes zero |
| molested | -0.1375 | -0.1638 | -0.1115 | 0.0009 | -0.0026 | 0.0046 | Includes zero |
| muslim | -0.0104 | -0.0385 | 0.0177 | -0.0008 | -0.0046 | 0.0031 | Includes zero |
| mute | -0.0385 | -0.0649 | -0.0119 | -0.0047 | -0.0084 | -0.0011 | Not zero |
| old | 0.1135 | 0.0860 | 0.1410 | -0.0065 | -0.0104 | -0.0026 | Not zero |
| paroled | -0.0391 | -0.0658 | -0.0120 | -0.0035 | -0.0072 | 0.0001 | Includes zero |
| pierced | -0.0527 | -0.0792 | -0.0266 | -0.0043 | -0.0080 | -0.0007 | Not zero |
| polygamous | -0.1120 | -0.1396 | -0.0850 | 0.0009 | -0.0027 | 0.0047 | Includes zero |
| poor | -0.0607 | -0.0872 | -0.0343 | -0.0022 | -0.0057 | 0.0014 | Includes zero |
| prostitute | -0.1050 | -0.1322 | -0.0782 | 0.0014 | -0.0022 | 0.0051 | Includes zero |
| retarded | -0.1108 | -0.1372 | -0.0845 | 0.0013 | -0.0023 | 0.0049 | Includes zero |
| scarred | -0.1479 | -0.1749 | -0.1215 | 0.0011 | -0.0025 | 0.0048 | Includes zero |
| server | 0.0210 | -0.0067 | 0.0488 | -0.0087 | -0.0126 | -0.0050 | Not zero |
| short | -0.0893 | -0.1163 | -0.0622 | 0.0003 | -0.0033 | 0.0040 | Includes zero |
| smoker | -0.0318 | -0.0580 | -0.0048 | -0.0051 | -0.0088 | -0.0016 | Not zero |
| unattractive | -0.1569 | -0.1834 | -0.1303 | 0.0015 | -0.0020 | 0.0052 | Includes zero |
| uneducated | -0.0674 | -0.0933 | -0.0410 | -0.0022 | -0.0057 | 0.0013 | Includes zero |
| unemployed | -0.1009 | -0.1272 | -0.0747 | -0.0002 | -0.0037 | 0.0035 | Includes zero |
| wheelchair | -0.0730 | -0.0991 | -0.0467 | -0.0016 | -0.0051 | 0.0019 | Includes zero |

# Replication without health-related groups

Inspection of the trait stereotypes associated with the stigmatized groups revealed that a set of 8 health-related groups – cancer, diabetic, heartattack, herpes, hiv, psoriasis, std, stroke – had top trait associates that referred to polysemous health meanings of trait words (e.g., patient, severe) rather than the social trait stereotype meanings (e.g., “patient” referring to tolerant or accepting; “severe” referring to strict and harsh). Moreover, many of these groups had overlapping content (e.g., many revealed similar associates of traits like patient and severe). As such, to ensure our results were not spuriously influenced by including these groups, we recomputed all aggregate results after excluding these 8 health-related groups (as this would ensure we weren’t concluding stability only because of stability in health-related associations).

Despite excluding an entire set of groups, results were again similar to our primary analysis across all 58 groups. The intercept was negative, *b* = -0.062, 95% HDI = [-0.082, -0.043], and the slope on aggregate remained weak with only slight movement towards more negative representations across time, *b* = -0.0027, 95% HDI = [-0.0041, -0.0014], with 100% of the posterior estimates still falling within the ROPE. Furthermore, as before, most groups (29/50, or 58% of groups) had highest density intervals (HDIs) that included zero, indicating the relatively greater prevalence of the reproducibility mechanism.

# Controlling for polysemy, frequency, and semantic drift

Here, we explore whether other metrics of language transformation and evolution could account for the observed patterns of change and stability across stigmatized groups. Specifically, we focus on the factors of how groups’ labels vary in terms of their: (1) polysemy (multiple meanings), (2) frequency, and (3) semantic drift (changing meanings).

We relied on scores of these three factors computed by Hamilton and colleagues (2016). First, for polysemy, scores were calculated by assessing the “context diversity” of each word or, in essence, the extent to which a word’s neighbors were also neighbors of each other (more details in Hamilton and colleagues (2016), section 4.4). If a word has neighbors that are also neighbors of each other then it can be said to have low diversity, and therefore also a specific, non-polysemous representation. In contrast, if a word has neighbors that themselves have unique and different neighbors, then the word can be said to have a “diffuse” representation with high context diversity, and therefore high polysemy.

Second, for frequency, the scores are relative frequencies of the top 10,000-most frequent words in Google Books, which indicate how frequent a given word is relative to all other 10,000 words. Low frequencies can create word embeddings that are essentially “overfit” to one or two instances of the word. As such, rare words may look like outliers because they haven’t averaged across many observations that would stabilize the meaning. Thus, examining the frequency of words ensures that our results are not an artifact of groups with labels that are rare words.

Third and finally, semantic drift scores essentially capture the changes in the local neighborhoods of words (e.g., the word *gay* had drifted from having neighbors *daft, sweet, cheerful* in 1900 to having neighbors *lesbian, homosexual, bisexual* in 1990). Examining semantic drift matters in the current work because our primary question is about whether and how negative stereotypes of groups have shifted over time (i.e., whether a group term has become associated with more negative traits). To properly answer such questions, we need to examine stereotype change above and beyond how the group labels themselves may have shifted in their “dictionary definition” (or general semantic meaning). To calculate semantic drift scores, Hamilton and colleagues (2016) first selected the top-10,000 most frequent words. Second, two vectors of cosine similarities were calculated: one vector for all cosine similarities between a target word (e.g., *gay*) and its *k* nearest neighbors in year *t*; and a similar vector for the year *t*+*n*, where *n* would be the index of a final decade (e.g., 1990). Third, the cosine distance between the two vectors (at *t* and *t+n*) was computed to operationalize semantic drift over the decades *t* and *t+n* (e.g., 1900 and 1990). Higher scores indicate greater semantic drift (i.e., greater change in the word’s local neighborhood, essentially used as a proxy for the term’s “dictionary definition”).

Although only 21 groups had these additional scores of polysemy, frequency, and semantic drift, we performed a robustness analysis in which we calculated the average polysemy, frequency, and semantic drift of each group’s labels and then used those (z-scored) average scores as covariates in the Bayesian regression. Results, reported in Table S7, show: (1) the slope of time is still small and not meaningfully different from zero; and (2) there are no significant interactions between time and the three covariates, indicating that the covariates are not moderating the conclusions of how groups are changing. At the same time, results also suggest a meaningful main effect of frequency on latent valence (<5% of the posterior falls within the ROPE), such that more frequently-mentioned groups are more positive. However, because this result is not related to our primary interest in change, we leave interpretation of this result to future research.

**Table S7**.

Bayesian mixed effects model predicting valence from time while controlling for frequency, semantic drift, and polysemy: Population (aggregate) estimates

|  | ***Estimate*** | ***Estimate Error*** | ***Lower 95% HDI*** | ***Upper 95% HDI*** | ***% in ROPE***  ***(0.1*SD_y_)*** |
| --- | --- | --- | --- | --- | --- |
| ***Intercept*** | -0.0673 | 0.0151 | -0.0980 | -0.0388 | 0% |
| **Time** | -0.0019 | 0.0011 | -0.0041 | 0.0003 | 100% |
| **Frequency** | 0.0335 | 0.0170 | -0.0014 | 0.0660 | 2.82% |
| **Semantic Drift** | 0.0071 | 0.0159 | -0.0236 | 0.0388 | 25.34% |
| **Polysemy** | 0.0129 | 0.0165 | -0.0192 | 0.0451 | 19.16% |
| **Time X Frequency** | -0.0002 | 0.0012 | -0.0026 | 0.0023 | 100% |
| **Time X Drift** | -0.0007 | 0.0011 | -0.0030 | 0.0015 | 100% |
| **Time X Polysemy** | 0.0003 | 0.0012 | -0.0021 | 0.0026 | 100% |

# Full table of estimated change across groups

Table 1 in the main text provides Bayesian estimated random effects only for the latent valence (negativity) slopes; here, we also provide results for the estimated random effects for latent warmth and competence. Furthermore, we provide the *inverse* of the average cosine similarity between the top-10 traits across successive decades; we take the inverse of cosine similarities, such that higher scores will indicate more change (because it indicates more different top trait representations across time). To assist inference about the different patterns that each group follows, cosine similarities are categorized into quartiles, labelled as least change (0.69, 0.75), less change (0.75, 0.79), some change (0.79, 0.82), or most change (0.82, 0.85). Additionally, for classifications of top-trait change versus stability, we classify groups as showing top-trait change if they are in the top two quadrants (inverse cosine similarity > 0.79) or as showing top-trait stability if they are in the bottom two quadrants (inverse cosine similarity < 0.79). As described in the table note, rows in the table are shaded according to their classification within the six empirical patterns of the Stigma Stability Framework.

**Table S8.**

Change in latent warmth, latent competence, and top-10 trait semantic similarity for 58 groups across 100 years (1900-2000) of book text

| **Group** | **Change in**  **latent warmth** | **Change in**  **latent competence** | **Change in top traits** (Average inverse cosine similarity) |
| --- | --- | --- | --- |
| bipolar | 0.0004[-0.0022, 0.0030] | -0.0003[-0.0031, 0.0026] | 0.79 |
| blind | 0.0021[-0.0005, 0.0047] | 0.0005[-0.0023, 0.0034] | 0.73 |
| depressed | 0.0023[-0.0002, 0.0049] | 0.0003[-0.0025, 0.0032] | 0.71 |
| gang | 0.0024[-0.0002, 0.0051] | -0.0028[-0.0056, 0.0001] | 0.69 |
| heartattack | -0.0007[-0.0033, 0.0019] | -0.0012[-0.0039, 0.0017] | 0.78 |
| homeless | 0.0015[-0.0011, 0.0042] | -0.0002[-0.0031, 0.0027] | 0.72 |
| mute | 0.0001[-0.0025, 0.0028] | -0.0003[-0.0031, 0.0025] | 0.73 |
| prostitute | 0.0005[-0.0021, 0.0032] | -0.0026[-0.0055, 0.0002] | 0.71 |
| retarded | 0.0006[-0.0021, 0.0032] | -0.0012[-0.0040, 0.0017] | 0.78 |
| scarred | 0.0021[-0.0005, 0.0047] | -0.0005[-0.0034, 0.0024] | 0.74 |
| short | 0.0015[-0.0011, 0.0040] | -0.0018[-0.0045, 0.0009] | 0.75 |
| stroke | -0.0006[-0.0031, 0.0020] | -0.0008[-0.0035, 0.0020] | 0.79 |
| unattractive | 0.0025[-0.0001, 0.0053] | -0.0012[-0.0040, 0.0016] | 0.71 |
| abortion | 0.0002[-0.0024, 0.0027] | -0.0008[-0.0035, 0.0021] | 0.83 |
| alcoholic | 0.0012[-0.0013, 0.0037] | 0.0000[-0.0028, 0.0029] | 0.79 |
| black | 0.0024[-0.0001, 0.0050] | -0.0002[-0.0031, 0.0026] | 0.79 |
| cancer | -0.0007[-0.0033, 0.0019] | -0.0017[-0.0043, 0.0012] | 0.80 |
| divorced | 0.0001[-0.0024, 0.0028] | -0.0010[-0.0038, 0.0018] | 0.85 |
| herpes | -0.0005[-0.0031, 0.0021] | -0.0011[-0.0038, 0.0018] | 0.83 |
| molestor | 0.0005[-0.0021, 0.0031] | -0.0016[-0.0045, 0.0012] | 0.79 |
| muslim | 0.0024[-0.0004, 0.0052] | -0.0019[-0.0046, 0.0010] | 0.83 |
| schizophrenic | 0.0002[-0.0024, 0.0029] | -0.0006[-0.0033, 0.0022] | 0.83 |
| wheelchair | 0.0009[-0.0016, 0.0035] | -0.0005[-0.0033, 0.0023] | 0.79 |
| atheist | 0.0032[ 0.0005, 0.0058] | -0.0010[-0.0038, 0.0017] | 0.71 |
| criminal | 0.0039[ 0.0014, 0.0065] | 0.0004[-0.0024, 0.0032] | 0.74 |
| deaf | 0.0031[ 0.0005, 0.0058] | 0.0001[-0.0026, 0.0030] | 0.80 |
| disabled | 0.0029[ 0.0003, 0.0056] | 0.0005[-0.0022, 0.0033] | 0.79 |
| infertile | 0.0036[ 0.0009, 0.0063] | 0.0013[-0.0014, 0.0042] | 0.75 |
| middleeastern | 0.0034[ 0.0007, 0.0061] | -0.0016[-0.0044, 0.0012] | 0.79 |
| molested | 0.0038[ 0.0012, 0.0065] | -0.0008[-0.0035, 0.0019] | 0.75 |
| polygamous | -0.0009[-0.0036, 0.0016] | -0.0037[-0.0065,-0.0010] | 0.74 |
| poor | 0.0043[ 0.0017, 0.0070] | 0.0018[-0.0011, 0.0047] | 0.69 |
| unemployed | 0.0029[ 0.0003, 0.0055] | 0.0015[-0.0013, 0.0043] | 0.78 |
| asexual | -0.0081[-0.0110,-0.0054] | -0.0053[-0.0082,-0.0026] | 0.76 |
| aboriginal | -0.0022[-0.0050, 0.0004] | -0.0052[-0.0082,-0.0023] | 0.77 |
| asian | -0.0005[-0.0031, 0.0022] | -0.0029[-0.0056, 0.0000] | 0.85 |
| christian | -0.0022[-0.0048, 0.0005] | -0.0029[-0.0058, 0.0000] | 0.80 |
| dealer | -0.0018[-0.0045, 0.0008] | -0.0047[-0.0077,-0.0019] | 0.84 |
| fat | -0.0003[-0.0030, 0.0023] | -0.0028[-0.0055,-0.0001] | 0.77 |
| gay | -0.0038[-0.0065,-0.0011] | -0.0048[-0.0077,-0.0019] | 0.71 |
| hiv | -0.0075[-0.0103,-0.0047] | -0.0069[-0.0098,-0.0040] | 0.85 |
| indian | -0.0029[-0.0057,-0.0002] | -0.0042[-0.0071,-0.0014] | 0.79 |
| jewish | -0.0006[-0.0033, 0.0021] | -0.0025[-0.0052, 0.0003] | 0.80 |
| laborer | -0.0043[-0.0070,-0.0016] | -0.0060[-0.0088,-0.0031] | 0.76 |
| latino | -0.0028[-0.0054,-0.0001] | -0.0050[-0.0079,-0.0022] | 0.83 |
| old | -0.0032[-0.0059,-0.0005] | -0.0020[-0.0047, 0.0009] | 0.77 |
| paroled | -0.0010[-0.0036, 0.0016] | -0.0021[-0.0049, 0.0006] | 0.82 |
| pierced | -0.0026[-0.0053, 0.0000] | -0.0057[-0.0086,-0.0027] | 0.77 |
| psoriasis | -0.0018[-0.0044, 0.0008] | -0.0024[-0.0053, 0.0004] | 0.81 |
| server | -0.0033[-0.0060,-0.0007] | -0.0041[-0.0069,-0.0013] | 0.75 |
| std | -0.0024[-0.0049, 0.0001] | -0.0025[-0.0053, 0.0003] | 0.85 |
| transgender | -0.0056[-0.0082,-0.0030] | -0.0077[-0.0109,-0.0048] | 0.83 |
| uneducated | -0.0004[-0.0029, 0.0022] | -0.0020[-0.0047, 0.0008] | 0.69 |
| diabetic | -0.0019[-0.0046, 0.0007] | -0.0024[-0.0052, 0.0003] | 0.83 |
| drugaddict | -0.0006[-0.0033, 0.0020] | -0.0013[-0.0041, 0.0014] | 0.80 |
| immigrant | 0.0003[-0.0022, 0.0029] | -0.0017[-0.0044, 0.0011] | 0.83 |
| multiracial | -0.0003[-0.0028, 0.0024] | -0.0014[-0.0041, 0.0014] | 0.83 |
| smoker | 0.0003[-0.0023, 0.0028] | -0.0017[-0.0043, 0.0010] | 0.81 |

***Note.*** Groups are shaded according to their classified mechanism and empirical pattern. Blue shading indicates pattern 1 (deep stability), purple indicates pattern 2 (valence+semantic stability), green indicates pattern 3 (valence stability), brown indicates pattern 4 (replacement via semantics), orange indicates pattern 5 (replacement via non-semantic relations), red indicates pattern 6 (generalization).

**References**

Charlesworth, T. E. S., Caliskan, A., & Banaji, M. R. (2022a). Historical Representations of Social Groups Across 200 Years of Word Embeddings from Google Books. *Proceedings of the National Academy of Sciences*, *119*(28). https://doi.org/https://doi.org/10.1073/pnas.2121798119

Charlesworth, T. E. S., Caliskan, A., & Banaji, M. R. (2022b). Historical Representations of Social Groups Across 200 Years of Word Embeddings from Google Books. *Proceedings of the National Academy of Sciences*, *119*(28). https://doi.org/https://doi.org/10.1073/pnas.2121798119

Charlesworth, T. E. S., Sanjeev, N., Hatzenbuehler, M. L., & Banaji, M. R. (2023). Identifying and predicting stereotype change across 72 groups, four text sources, and historical time (1900-2015): Insights from word embeddings. *Journal of Personality and Social Psychology*, *125*(5), 969–990. https://doi.org/https://doi.org/10.1037/pspa0000354

Charlesworth, T. E. S., Yang, V., Mann, T. C., Kurdi, B., & Banaji, M. R. (2021). Gender Stereotypes in Natural Language: Word Embeddings Show Robust Consistency Across Child and Adult Language Corpora of More Than 65 Million Words. *Psychological Science*, *32*(2), 218–240. https://doi.org/10.1177/0956797620963619

Fiske, S. T., Cuddy, A. J. C., Glick, P., & Xu, J. (2002). A model of (often mixed) stereotype content: Competence and warmth respectively follow from perceived status and competition. *Journal of Personality and Social Psychology*, *82*(6), 878–902. https://doi.org/10.1037//0022-3514.82.6.878

Hamilton, W. L., Leskovec, J., & Jurafsky, D. (2016). Diachronic word embeddings reveal statistical laws of semantic change. *54th Annual Meeting of the Association for Computational Linguistics, ACL 2016 - Long Papers*, *3*, 1489–1501. https://doi.org/10.18653/v1/p16-1141

Kruschke, J. K. (2018). Rejecting or Accepting Parameter Values in Bayesian Estimation. *Advances in Methods and Practices in Psychological Science*, *1*(2), 270–280. https://doi.org/10.1177/2515245918771304

Nicolas, G., Bai, X., & Fiske, S. T. (2021). Comprehensive stereotype content dictionaries using a semi-automated method. *European Journal of Social Psychology*, *51*(1), 178–196. https://doi.org/10.1002/EJSP.2724/FORMAT/PDF

Pachankis, J. E., Hatzenbuehler, M. L., Wang, K., Burton, C. L., Crawford, F. W., Phelan, J. C., & Link, B. G. (2017). The Burden of Stigma on Health and Well-Being: A Taxonomy of Concealment, Course, Disruptiveness, Aesthetics, Origin, and Peril Across 93 Stigmas. *Personality and Social Psychology Bulletin*, 014616721774131. https://doi.org/10.1177/0146167217741313
